# Clicked Cinnamic/Caffeic Esters and Amides as Radical Scavengers and 5-Lipoxygenase Inhibitors

**DOI:** 10.1155/2014/931756

**Published:** 2014-02-18

**Authors:** Jérémie A. Doiron, Benoît Métayer, Ryan R. Richard, Dany Desjardins, Luc H. Boudreau, Natalie A. Levesque, Jacques Jean-François, Samuel J. Poirier, Marc E. Surette, Mohamed Touaibia

**Affiliations:** ^1^Département de Chimie et Biochimie, Université de Moncton, Moncton, NB, Canada E1A 3E9; ^2^Centre de Recherche en Rhumatologie et Immunologie et Faculté de Médecine, Université Laval, Québec, QC, Canada G1V 4G2

## Abstract

5-Lipoxygenase (5-LO) is the key enzyme responsible for the conversion of arachidonic acid to leukotrienes, a class of lipid mediators implicated in inflammatory disorders. In this paper, we describe the design, synthesis, and preliminary activity studies of novel clicked caffeic esters and amides as radical scavengers and 5-LO inhibitors. From known 5-LO inhibitor **3** as a lead, cinnamic esters **8a–h** and amides **9a–h** as well as caffeic esters **15a–h** and amides **16a–h** were synthesized by Cu(I)-catalyzed [1,3]-dipolar cycloaddition with the appropriate azide precursors and terminal alkynes. All caffeic analogs are proved to be good radical scavengers (IC_50_: 10–20 *μ*M). Esters **15g** and **15f** possessed excellent 5-LO inhibition activity in HEK293 cells and were equipotent with the known 5-LO inhibitor CAPE and more potent than Zileuton. Several synthesized esters possess activities rivaling Zileuton in stimulated human polymorphonuclear leukocytes.

## 1. Introduction

It has been firmly established that leukotrienes (LTs), eicosanoid inflammatory mediators derived from arachidonic acid (AA), play a key role in inflammatory and allergic responses [1]. Further findings show probable links to a large range of physiological disorders such as cancer, atherosclerosis, asthma, and irritable bowel syndrome, amongst others [2].

The key transformation towards LTs biosynthesis is the initial conversion of AA to leukotriene A_4_ (LTA_4_) catalyzed by 5-lipoxygenase (5-LO). Following appropriate cellular stimulation, intracellular calcium influx and phosphorylation of 5-LO stimulate the translocation of this enzyme from the cytosol to the nuclear membrane, where it associates with the membrane-bound 5-LO activating protein (FLAP). Upon release of phospholipid-bound AA by cytosolic phospholipase A_2_ (cPLA_2_), AA is transferred to 5-LO by FLAP. 5-LO catalyzes the oxygenation of AA with molecular oxygen yielding the unstable eicosanoid hydroperoxyde 5-HpETE, which then converts to leukotriene epoxide LTA_4_. Subsequent enzymatic hydrolysis of LTA_4_ yields LTB_4_, a potent chemotactic and chemokinetic mediator [2]. Alternatively, LTA_4_ can be converted by the action of LTC_4_-synthase to cysteinyl leukotrienes LTC_4_, LTD_4_, and LTE_4_, which are assumed to play important roles not only in asthma and allergic rhinitis, but also in chronic inflammation and in the regulation of the adaptive immune response [2].

Anti-LTs therapy, either by the use of LT receptor antagonists or by the use of 5-LO or FLAP inhibitors, improves pulmonary function and decreases symptoms and severity of asthma [3–5] and improves nasal congestion and rhinorrhea in allergic rhinitis [6–8]. Severity of atherosclerotic lesions and plaque instability correlate with 5-LO levels [9], with both LTB_4_ and the cysteinyl LTs involved in atherosclerosis development [10]. Additionally, high expression of 5-LO has been measured in numerous cancerous cells lines, namely, in colorectal, esophageal, and pancreatic adenocarcinomas as well as in melanoma, lymphoma, and leukemia cell lines [11, 12]. Inhibition of LTs biosynthesis has been shown to reduce the incidence and volume of tumors in certain models of lung cancer [13], gastroesophageal adenocarcinoma [14], and squamous cell carcinoma [15], while inhibition of 5-LO was shown to induce massive apoptosis in prostate cancer cell lines [16].

**Figure 1:**
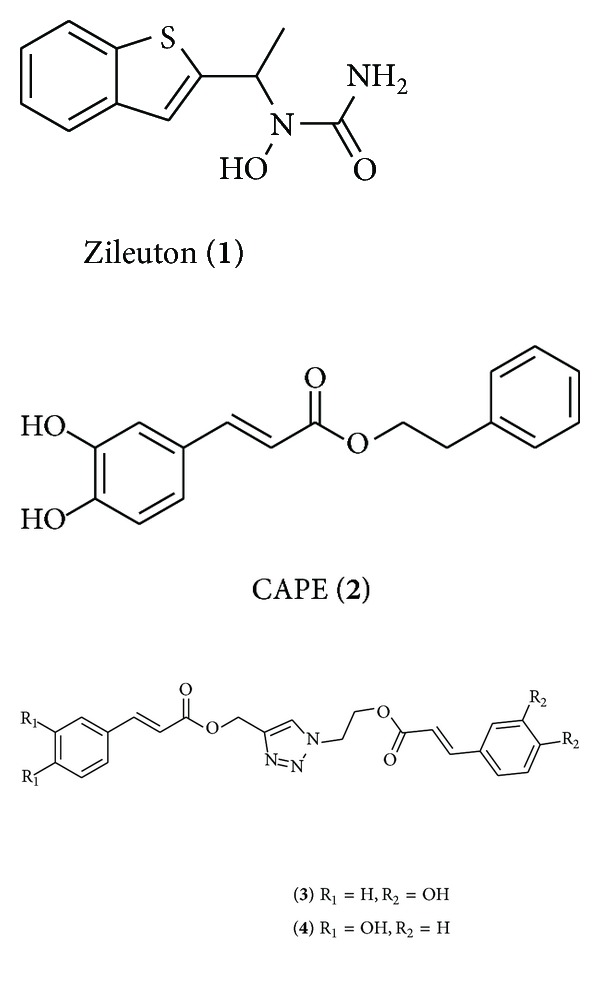
Structure of some known 5-LO inhibitors.

Given the biological importance of LTs in various pathologies, it is not surprising that the development of efficient inhibitors of their biosynthesis has been the subject of much interest, with 5-LO being an ideal target for complete inhibition of LTs synthesis [17]. Among known inhibitors, only the antiasthmatic drug Zileuton (**1**, Figure 1) is approved for human use as the racemic mixture [2, 18]. However, undesirable hepatotoxicity as well as its pharmacokinetic profile requiring frequent and large dosing limits its usefulness in treatments [2, 18–20].

Natural polyphenolic compounds derived from caffeic acids such as CAPE (**2**) (Figure 1, caffeic acid phenethyl ester) and chicoric acid have been thoroughly investigated for their diverse bioactivities, including antioxidant, radical scavenging, anticancer, antibacterial, and antiviral properties [21]. Both caffeic acid and CAPE (**2**) have been evaluated for lipoxygenase inhibition potentials [22–25], with CAPE (**2**) showing 5-LO inhibition equaling the inhibitory power of Zileuton (**1**) in whole blood and surpassing Zileuton (**1**) in stimulated polymorphonuclear leukocytes (PMNL) [26]. In our search for efficient inhibitors of 5-LO, a study was undertaken to probe the effects of multivalency on caffeic ester derivatives for 5-LO inhibition [27, 28]. A series of multivalent caffeic esters bearing 1–6 caffeoyl or cinnamoyl ester moieties were synthesized and evaluated for inhibitory capacity in lysed as well as whole HEK293 cell cultures. Though multivalency was not found to play a significant role in inhibitory capacity of these compounds (IC_50_/caffeoyl residue did not reflect the number of caffeoyl esters in each compound), several lead compounds were found to exhibit excellent inhibitory power for 5-LO. Notable are clicked caffeic/cinnamic derivatives **3** and **4 **(Figure 1), each bearing a single caffeoyl residue tied to the analogous cinnamoyl residue by a 1,4-disubstituted-1,2,3-triazole core. Compound** 3**, with an IC_50_ of 0.68 *μ*M in cell lysates, rivals Zileuton (**1**) (IC_50_ = 0.5–1 *μ*M) for inhibitory power [29], and compound **4** induced near complete 5-LO inhibition in whole cells at 10 *μ*M, surpassing Zileuton (**1**). This study also firmly highlighted the importance of the 3,4-dihydroxylated aromatic ring of the caffeoyl pharmacophore for inhibitory activity, as all cinnamic analogs failed to produce notable inhibition at reasonable concentrations.

In an effort to further explore the possibilities offered by the lead monomeric caffeoyl triazole compound **3**, four new series of compounds bearing similar structural motifs and conserving the important caffeoyl residue were synthesized and tested for 5-LO inhibition. As the inhibitory activity of caffeoyl inhibitors is thought to stem from a combination of lipophilic interactions, iron chelation by the catechol moiety, and antioxidant/radical scavenging interactions with 5-LO catalytic ferric iron atom, radical scavenging tests have also been performed [30–32]. The choice to specifically pursue investigation of lead compound **3** over compound** 4**, differing only by the relative orientation of the 1-ethyl-4-methyl-1H-1,2,3-triazole bridge linking the terminal ester moieties, was motivated by the ready commercial availability of aryl acetylenes.

## 2. Materials and Methods

### 2.1. Chemistry

All chemicals used were purchased from Aldrich (CA) and used without further purification. Purification of compounds was carried out by silica gel circular chromatography (Chromatotron model 7924, Harrison Research) or by flash chromatography. TLC was run on silica gel coated aluminium sheets (SiliaPlate TLC, Silicycle) with detection by UV light (254 nm, UVS-11, Mineralight shortwave UV lamp). Melting points were obtained using a MELTEMP (model 1001D) melting point apparatus. FTIR spectra were recorded on a Nicolet Impact 400 spectrometer. NMR spectra were recorded on a Bruker Avance III 400 MHz spectrometer using TMS as an internal standard. High-resolution mass measurements were performed on a Bruker Daltonics' micrOTOF instrument in positive or negative electrospray.

### 2.2. General Procedure I: Monosubstituted Triazoles from Organic Azides and Acetylene

To a vigorously stirred solution of the appropriate organic azide (1 mmol, 1 eq.) in 6 mL DMSO is added copper (I) iodide (0.1 mmol, 0.1 eq.), after which the reaction vessel is thoroughly flushed with acetylene gas and sealed under balloon pressure. Triethylamine (1.2 mmol, 1.2 eq.) is then added and the mixture is left to react overnight at room temperature. The resulting solution is partitioned between 125 mL of brine and 25 mL ethyl acetate, after which the aqueous phase is extracted three more times with 25 mL ethyl acetate. The organic phase is then washed twice with brine, treated with charcoal, dried over MgSO_4_, and concentrated. The resulting oil is purified by silica gel circular chromatography (Chromatotron model 7924, Harrison Research, eluent: MeOH/CH_2_Cl_2_).

### 2.3. General Procedure IIA: Anhydrous CuAAC Reaction

To a vigorously stirred solution of the appropriate organic azide (0.5 mmol, 1 eq.) and alkyne (0.75 mmol, 1.5 eq.) in 4 mL THF is added copper (I) iodide (0.025 mmol, 0.05 eq.) followed by triethylamine (0.6 mmol, 1.2 eq.). The reaction mixture is stirred overnight under argon atmosphere. The resulting solution is partitioned between 30 mL AcOEt and 30 mL H_2_O, after which the aqueous phase is extracted twice more with 30 mL AcOEt. The combined organic fractions are washed twice with saturated ammonium chloride (20 mL), twice with brine, dried over MgSO_4_, and concentrated. The resulting oil is purified by silica gel circular chromatography (Chromatotron model 7924, Harrison Research, eluent: AcOEt/Hex or MeOH/CH_2_Cl_2_).

### 2.4. General Procedure IIB: Aqueous CuAAC Reaction

To a vigorously stirred solution of the appropriate organic azide (0.5 mmol, 1 eq.) and alkyne (0.75 mmol, 1.5 eq.) in 2.5 mL THF is added CuSO_4_ *·*5H_2_O (0.05 mmol, 0.1 eq.) dissolved in 2.5 mL H_2_O followed by sodium ascorbate (0.05 mmol, 0.1 eq.), after which the mixture is left to react overnight. The resulting solution is then diluted to 30 mL with water and extracted three times with AcOEt (20 mL). The organic fractions are then combined, washed twice with water, twice with saturated ammonium chloride, twice with brine, dried over MgSO_4_, and concentrated. The resulting oil is purified by silica gel circular chromatography (Chromatotron model 7924, Harrison Research, eluent: AcOEt/Hex or MeOH/CH_2_Cl_2_).

### 2.5. General Procedure III: Deacetylation of Diacetylcaffeoyl Derivatives

The appropriate diacetylcaffeoyl derivative (0.25 mmol, 1 eq.) is dissolved in 2 mL anhydrous CH_2_Cl_2_ under N_2_, to which is added 4 mL MeOH. To the resulting stirred solution is added guanidinium hydrochloride (0.81 mmol, 3.25 eq.) followed by triethylamine (2.44 mmol, 9.75 eq.). After consumption of the diacetylated precursor (about 2 h), the reaction mixture is concentrated and partitioned between 60 mL of AcOEt and 30 mL of water. The organic phase is then washed again with water, twice with saturated ammonium chloride, twice with brine, treated with charcoal, dried over MgSO_4_ and concentrated to give the resulting pure caffeoyl derivative.

### 2.6. 2-Azidoethyl Cinnamate **(6)**

A mixture of cinnamic acid (1000 mg, 3.78 mmol, 1 eq.), 3-4 drops of anhydrous DMF, and 20 mL of thionyl chloride was heated at reflux for 3 h, under Ar. The excess thionyl chloride was then evaporated at reduced pressure, and the residue dissolved in 5 mL of CH_2_Cl_2_. The resulting solution was added dropwise to a mixture of 2-azidoethanol (1315 mg, 15.1 mmol, 4 eq.) and pyridine (1255 mg, 15.87 mmol, 4.2 eq.) in 15 mL of CH_2_Cl_2_ at 0°C under argon, then left to return to ambient temperature overnight. After concentration, the oily residue was diluted with 90 mL AcOEt and washed with 2 × 30 mL H_2_O, 2 × 30 mL NH_4_Cl_sat_, 2 × NaCl_sat_, dried over MgSO_4_, filtered, and concentrated. Compound **6** (1040 mg, 4.8 mmol) was obtained as yellow oil after silica gel circular chromatography (0–15% AcoEt/Hex), yield = 72%. Rf = 0.29 (20% AcOEt/Hex). ^1^H NMR (400 MHz, CDCl_3_, 25°C), *δ* (ppm) = 7.76 (d, 1H, *J* = 16.0 Hz, =CHC_ar_), 7.58–7.54 (m, 2H, H_ar_), and 7.44–7.42 (m, 3H, H_ar_), 6.49 (d, 1H, *J* = 16.0 Hz, =CHCO), 4.41 (t, 2H, *J* = 5.1 Hz, CH_2_OCO), 3,58 (t, 2H, *J* = 5.1 Hz, CH_2_N_3_); ^13^C NMR (101 MHz, CDCl_3_, 25°C), and *δ* (ppm) = 166.6, 145.8, 134.2, 130.6, 129.0, 128.2, 117.2, 63.1, 49.9. HRMS *m*/*z* calc. for C_11_H_11_N_3_O_2_ + (H^+^): 218.0929; found: 218.0922.

### 2.7. N-(2-Azidoethyl)cinnamamide **(7)**

To a stirred solution of cinnamic acid (400 mg, 2.76 mmol) in 4 mL anhydrous CH_2_Cl_2_ at 0°C and under argon was added 3-4 drops of anhydrous DMF followed by dropwise addition of oxalyl chloride (700 mg, 5.52 mmol, 2 eq.). After 3 h, the resulting solution was concentrated with a stream of dry nitrogen, re-dissolved in CH_2_Cl_2_, and brought to dryness once more with nitrogen to yield the acyl chloride as an oily solid. To a stirred solution of 2-azidoethanamine (238 mg, 2.76 mmol, 1 eq.) in 3 mL CH_2_Cl_2_ containing pyridine (218 mg, 2.76 mmol, 1 eq.) was added dropwise the acyl chloride, dissolved in 2 mL CH_2_Cl_2_, while keeping the solution at 0°C and under argon. The solution was left to return to room temperature overnight, after which the mixture was diluted to 75 mL with CH_2_Cl_2_, washed with 2 × 30 mL H_2_O, 2 × 30 mL NH_4_Cl_sat_, 2 × NaCl_sat_, dried over MgSO_4_, filtered, and concentrated. Compound **7** was obtained as a yellow oil after silica gel circular chromatography (0-1% MeOH/CH_2_Cl_2_), yield = 70%. Rf = 0.53 (6% MeOH/CH_2_Cl_2_). ^1^H NMR (400 MHz, CDCl_3_, 25°C), *δ* (ppm) = 7.67 (d, 1H, *J* = 15.6 Hz, =CHC_ar_), 7.51-7.50 (m, 2H, H_ar_), 7.39–7.34 (m, 3H, H_ar_), 6.56 (d, 1H, *J* = 15.6 Hz, =CHCO), 6.22 (br s, 1H, NH), 3.60–3.50 (m, 4H, CH_2_CH_2_). ^13^C NMR (101 MHz, CDCl_3_, 25°C), *δ* (ppm) = 166.22, 141.65, 134.65, 129.86, 129.34, 128.85, 127.86, 127.07, 121.15, 50.97, 39.09. HRMS *m*/*z* calc. for C_11_H_12_N_4_O + (H^+^): 217.1084; detected: 217.1084.

### 2.8. 2-(1H-1,2,3-Triazol-1-yl)ethyl Cinnamate **(8a)**

Following general procedure I with azide **6, **compound **8a** was obtained as a white powder after silica gel circular chromatography (1% MeOH/CH_2_Cl_2_), yield = 88%. Mp = 99-100°C, *R*_*f*_ = 0.37 (5% MeOH/CH_2_Cl_2_). ^1^H NMR (400 MHz, CDCl_3_, 25°C), *δ* (ppm) = 7.76 (s, 1H, =CHN), 7.70 (d, 1H, *J* = 16.5 Hz, =CHC_ar_), 7.68 (*s*, 1H, =CHN), 7.55–7.52 (m, 2H, H_ar_), 7.42 (t, 3H, H_ar_), 6.42 (d, 1H, *J* = 16.0 Hz, =CHCO), 4.76 (t, 2H, *J* = 5.0 Hz, OCH_2_), 4.64 (t, 2H, *J* = 5.4 Hz, CH_2_N). ^13^C NMR (101 MHz, CDCl_3_, 25°C), *δ* (ppm) = 168.29, 146.21, 134.10, 133.98, 130.73, 128.98, 128.24, 124.01, 116.76, 62.52, 49.04. HRMS *m*/*z* calc. for C_13_H_13_N_3_O_4_ + (H^+^): 244.1086; detected: 244.1091.

### 2.9. 2-(4-Propyl-1H-1,2,3-triazol-1-yl)ethyl Cinnamate **(8b)**

Following general procedure IIA with azide **6 **and 1-pentyne, compound **8b** was obtained as a white crystals after silica gel circular chromatography (0–35% AcOEt/Hex), yield = 70%. Mp = 63-64°C, *R*_*f*_ = 0.50 (50% AcOEt/Hex). ^1^H NMR (400 MHz, CDCl_3_, 25°C), *δ* (ppm) = 7.71 (d, 1H, *J* = 16.0 Hz, =CHC_ar_), 7.55–7.52 (m, 2H, H_ar_), 7.43–7.38 (m, 4H, H_ar_ + =CHN), 6.43 (d, 1H, *J* = 16.0 Hz, =CHCO), 4.68 (t, 2H, *J* = 5.3 Hz, OCH_2_), 4.62 (t, 2H, *J* = 5.2 Hz, CH_2_N), 2.73 (t, 2H, *J* = 7.6 Hz, =CCH_2_), 1.72 (m, 2H, C**H**_**2**_CH_3_), 0.98 (t, 3H, *J*= 7.4 Hz, CH_3_); ^13^C NMR (101 MHz, CDCl_3_, 25°C), *δ* (ppm) = 166.3, 148.5, 146.1, 134.0, 130.7, 129.0, 128.2, 121.2, 116.9, 62.6, 49.0, 27.7, 22.7, 13.8. HRMS *m*/*z* calc. for C_16_H_19_N_3_O_2_ + H^+^: 186.1550; detected: 286.1543.

### 2.10. (E)-4-(3-(2-Azidoethoxy)-3-oxoprop-1-en-1-yl)-1,2-phenylene Diacetate **(11)**

Following the same procedure as **6**, but with diacetylcaffeic acid **10 **instead of cinnamic acid **1**, compound **11** was obtained as white crystals after silica gel circular chromatography (0–30% AcOEt/Hex), yield = 65%. Mp = 81–84°C, *R*_*f*_ = 0.27 (30% AcOEt/Hex). ^1^H NMR (400 MHz, CDCl_3_, 25°C), *δ* (ppm) = 7.69 (d, 1H, *J*= 16.0 Hz, =CHC_ar_), 7.44 (dd, 1H, *J*= 8.4 Hz, 2.0 Hz, H_ar_), 7.39 (d, 1H, *J* = 2.0 Hz, H_ar_), 7.26 (d, 1H, *J* = 8.4 Hz, H_ar_), 6.43 (d, 1H, *J* = 16.0 Hz, =CHCO), 4.40 (t, 2H, *J* = 5.2 Hz, OCH_2_), 3.58 (t, 2H, *J* = 5.0 Hz, CH_2_N), 2.33 (s, 3H, CH_3_COO), 2.32 (s, 3H, CH_3_COO); ^13^C NMR (101 MHz, CDCl_3_, 25°C), *δ* (ppm) = 168.1, 168.0, 166.1, 143.9, 143.7, 142.5, 133.1, 126.6, 124.0, 122.9, 118.4, 63.2, 49.9, 20.7, 20.6. HRMS *m*/*z* calc. for C_15_H_15_O_6_N_3_ + (H^+^): 334.1039; found: 334.1033.

### 2.11. (E)-4-(3-((2-Azidoethyl)amino)-3-oxoprop-1-en-1-yl)-1,2-phenylene Diacetate **(12)**

Following the same procedure as **7**, but with diacetylcaffeic acid **10 **instead of cinnamic acid **1**, compound **12** was obtained as a white solid after silica gel circular chromatography (0-1% MeOH/CH_2_Cl_2_), yield = 71%. Mp = 97-98°C, *R*_*f*_ = 0.55 (5% MeOH/CH_2_Cl_2_). ^1^H NMR (400 MHz, CDCl_3_, 25°C), *δ* (ppm) = 7.58 (d, 1H, *J* = 15.6 Hz, =CHC_ar_), 7.38 (dd, 1H, *J* = 8.4 Hz, 1.8 Hz, H_ar_), 7.35 (d, 1H, *J* = 1.8 Hz, H_ar_), 7.21 (d, 1H, *J* = 8.4 Hz, H_ar_), 6.34 (d, 1H, *J* = 15.6 Hz, =CHCO), 6.07 (m, 1H, NH), 3.59–3.51 (m, 4H, NCH_2_CH_2_N_3_), 2.33 (s, 3H, CH_3_COO), 2.32 (s, 3H, CH_3_COO). ^13^C NMR (101 MHz, CDCl_3_, 25°C), *δ* (ppm) = 168.16, 168.12, 165.66, 143.08, 142.38, 139.79, 133.64, 126.26, 123.85, 122.40, 121.31, 50.89, 39.07, 20.66, 20.64. HRMS *m*/*z* calc. for C_15_H_16_N_4_O_5_ + (H^+^): 333.1193; found: 333.1190.

### 2.12. (E)-4-(3-oxo-3-(2-(4-Propyl-1H-1,2,3-triazol-1-yl)ethoxy)prop-1-en-1-yl)-1,2-phenylene Diacetate **(13b)**

Following general procedure IIB with azide **11 **and 1-pentyne, compound **13b** was obtained as a white powder after silica gel circular chromatography (0–0.7% MeOH/CH_2_Cl_2_), yield = 73%. Mp = 100°C, *R*_*f*_ = 0.48 (4% MeOH/CH_2_Cl_2_). ^1^H NMR (400 MHz, CDCl_3_, 25°C), *δ* (ppm) = 7.64 (d, 1H, *J* = 16.0 Hz, =CHC (_ar_)), 7.42 (dd, 1H, *J* = 8.4 Hz, 1.9 Hz, H_ar_), 7.38 (d, 1H, *J* = 1.9 Hz, H_ar_), 7.39 (s, 1H, =CHN), 7.25 (d, 1H, *J* = 8.4 Hz), 6.37 (d, 1H, *J* = 16.0 Hz, =CHCO), 4.67 (t, 2H, *J* = 5.0 Hz, OCH_2_), 4.61 (t, 2H, *J* = 5.0 Hz, CH_2_N), 2.72 (t, 2H, *J* = 7.6 Hz, **CH**_**2**_CH_2_CH_3_), 2.34 (s, 3H, CH_3_COO), 2.33 (s, 3H, CH_3_COO), 1.77–1.69 (m, 2H, CH_2_ **CH**_**2**_CH_3_), 0.99 (t, 3H, *J* = 7.4 Hz). ^13^C NMR (101 MHz, CDCl_3_, 25°C), *δ* (ppm) = 168.09, 167.96, 165.93, 148.58, 144.12, 143.79, 142.50, 132.87, 126.61, 124.04, 122.84, 121.20, 118.05, 62.76, 48.94, 27.65, 22.73, 20.67, 20.63, 13.77. HRMS *m*/*z* calc. for C_20_H_23_N_3_O_6_ + H^+^: 402.1660; detected: 402.1656.

### 2.13. (E)-2-(1H-1,2,3-Triazol-1-yl)ethyl 3-(3,4-Dihydroxyphenyl)acrylate **(15a)**

Following general procedure III with acetylated caffeoyl derivative **13a, **compound **15a** was obtained as a white powder, yield = 81%. Mp = 196–198°C (dec.), *R*_*f*_ = 0.31 (5% MeOH/CH_2_Cl_2_). ^1^H NMR (400 MHz, DMSO-d_6_, 25°C), *δ* (ppm) = 9.6–9.1 (br s, 2H, OH), 8.21 (s, 1H, =CHN), 7.75 (s, 1H, =CHN), 7.44 (d, 1H, *J* = 15.9 Hz, =CHC_ar_), 7.04 (d, 1H, *J* = 1.8 Hz, H_ar_), 6.99 (dd, 1H, *J* = 8.2 Hz, 1.8 Hz, H_ar_), 6.76 (d, 1H, *J* = 8.2 Hz, H_ar_), 6.22 (d, 1H, *J* = 15.9 Hz, =CHCO), 4.72 (t, 2H, *J* = 5.1 Hz, OCH_2_), 4.51 (t, 2H, *J* = 5.1 Hz, CH_2_N). ^13^C NMR (101 MHz, DMSO-d_6_, 25°C), *δ* (ppm) = 166.57, 149.06, 146.24, 146.04, 133.84, 125.78, 125.74, 121.98, 116.19, 115.35, 113.67, 62.74, 48.91. HRMS *m*/*z* calc. for C_13_H_13_N_3_O_4_ + (H^+^): 276.0979; detected: 276.0985.

### 2.14. Biology/Biochemistry

#### 2.14.1. Antiradical Activity Assay

The radical scavenging activity of test compounds was measured as previously described using 2,2-diphenyl-1-picrylhydrazyl (DPPH) as a stable radical [25] with slight modifications. Particular care was taken in the preparation of the control (DPPH reagent + ethanol as a diluent without test compounds). Controls with O.D. of 0.350-0.360 at 520 nm were deemed as acceptable to avoid variations in IC_50_ calculations. 1 mL of DPPH in ethanol (60 mM) was mixed with 1 mL of the test compounds at the indicated concentrations or their diluent (ethanol). Each mixture was then shaken vigorously and held in the dark for 30 min at room temperature. The absorbance of DPPH at 520 nm was then measured. The radical scavenging activity was expressed in terms of % inhibition of DPPH absorbance:

(1) $$\begin{array}{c} \%\;\text{Inhibition}=\left[\frac{\left(A_{\text{control}}-A_{\text{test}}\right)}{\left(A_{\text{control}}\right)}\right]\;\;\times 100, \end{array}$$

where *A*_control_ is the absorbance of the control (DPPH solution without test compound) and *A*_test_ is the absorbance of the test sample (DPPH solution plus compound). Data are expressed as means ± SD of 2 independent experiments, each performed in triplicate (Table 1). IC_50_ values were calculated from a sigmoidal concentration-response curve fitting model with a variable slope on GraphPad Prism 5 software (GraphPad software, San Diego, California).

#### 2.14.2. 5-LO Products Biosynthesis Assays in HEK293 Cells

HEK293 cells were cotransfected with a pcDNA3.1 vector expressing 5-LO and a pBUDCE4.1 vector expressing 5-LO activating protein (FLAP) using Polyfect reagent (QIAGEN, Mississauga, ON, Canada) according to the manufacturer's protocol. Stable transfections of HEK293 cells were obtained following cell culture in the presence of Geneticin and Zeocin (Invitrogen, Burlington, ON, Canada). The resulting stable double transfectants were propagated in culture and aliquots were frozen. Once thawed for a series of experiments, each aliquot of cells is cultured for a maximum of 6 weeks before being discarded.

**Figure 2:**
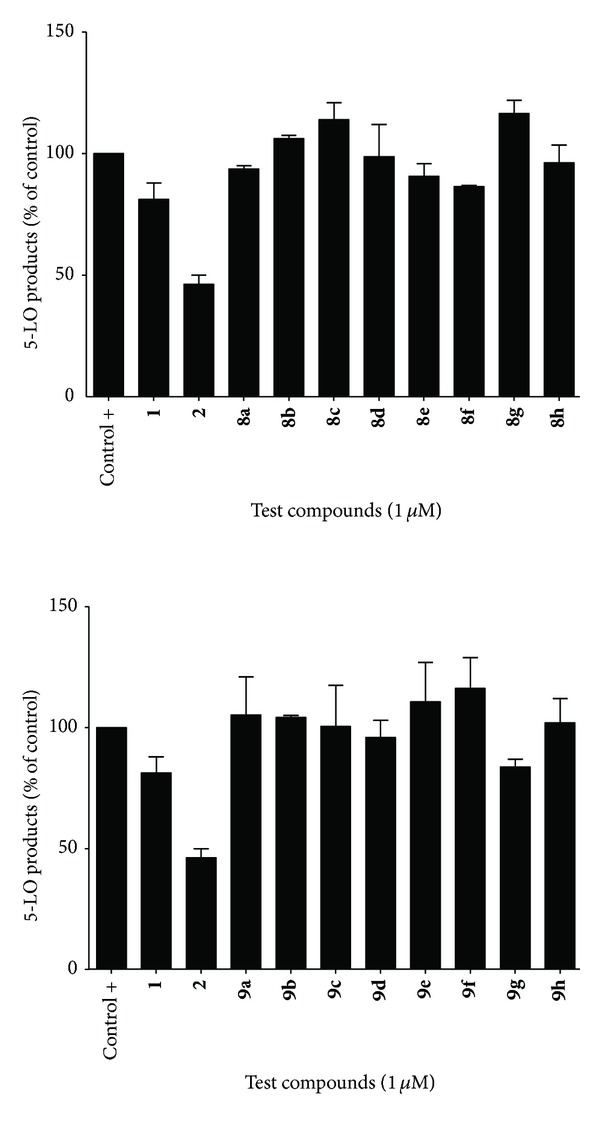
Inhibition of 5-LO products synthesis by cinnamic esters **(8a–h)** and amides **(9a–h)** in whole HEK293 cells.

**Figure 3:**
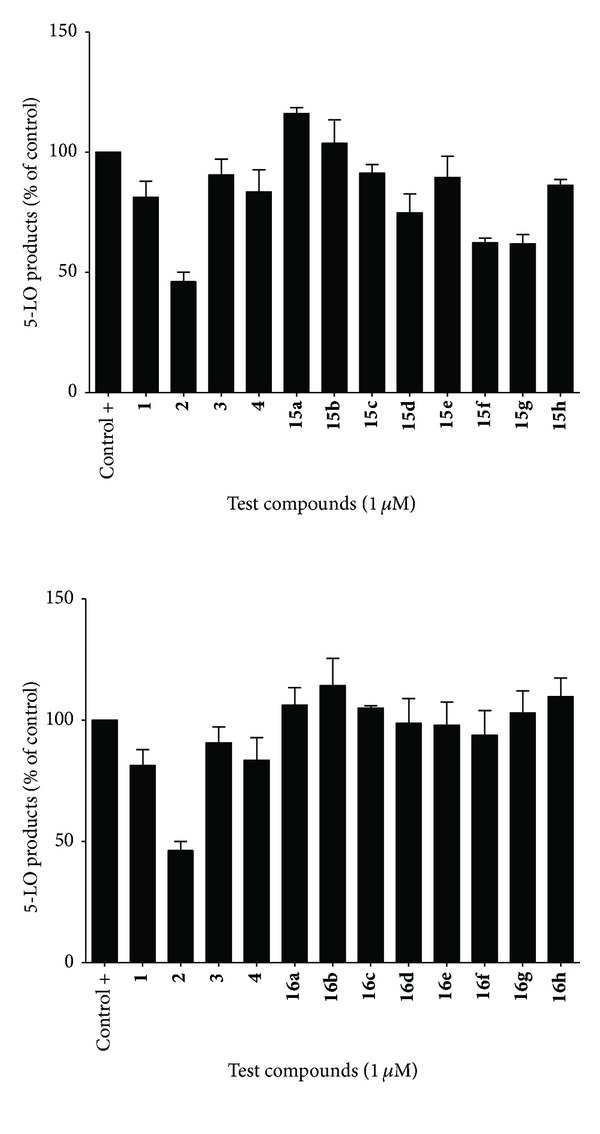
Inhibition of 5-LO products synthesis by caffeic esters (**15a–h**) and amides (**16a–h**) in whole HEK293 cells.

For cell stimulation of 5-LO products, transfected HEK293 cells were collected following trypsinization, washed and the cell pellet was resuspended in Hank's balanced salt solution (HBSS) (Lonza, Walkersville, MD) containing 1.6 mM CaCl_2_ at a concentration of 5 × 10^5^ cells mL^−1^. Cells were preincubated with each compound at the indicated concentration for 5 min at 37°C. Cells were then stimulated for 15 minutes at 37°C with the addition of 10 *μ*M calcium ionophore A23187 (Sigma-Aldrich, Oakville, ON, Canada) and 10 *μ*M arachidonic acid (Cayman Chemical, Ann Arbor, MI). Stimulations were stopped and processed on RP-HPLC as described previously [26, 27]. Data are expressed as means ± SEM of 3 independent experiments, each performed in duplicate (Figures 2 and 3).

#### 2.14.3. 5-LO Products Biosynthesis Assays in Human PMNL Cells

**Figure 4:**
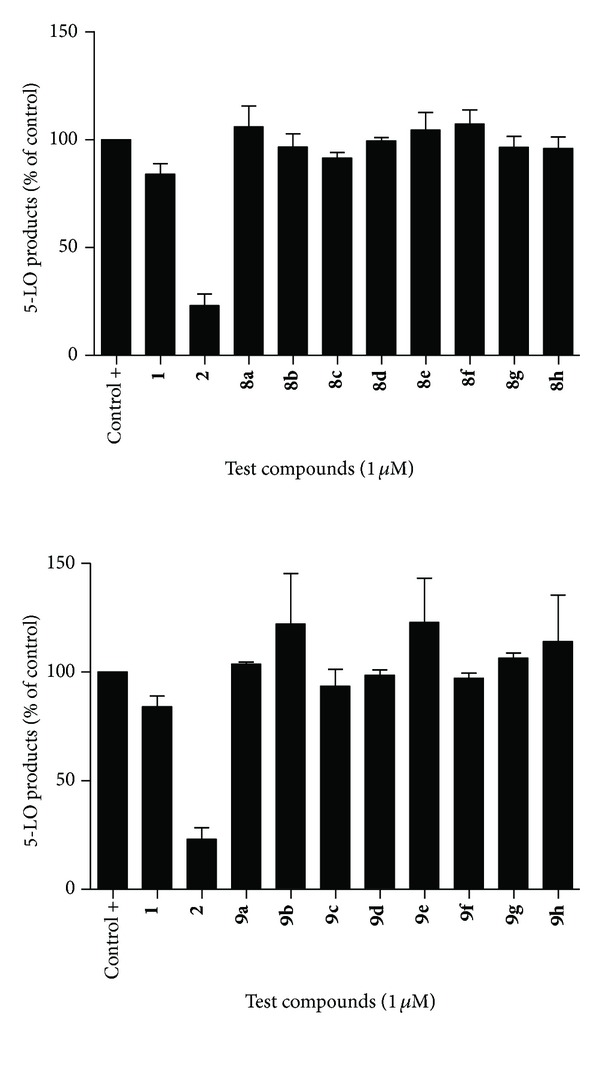
Inhibition of 5-LO products synthesis by cinnamic esters (**8a–h**) and amides (**9a–h**) in thapsigargin stimulated human PMNL.

**Figure 5:**
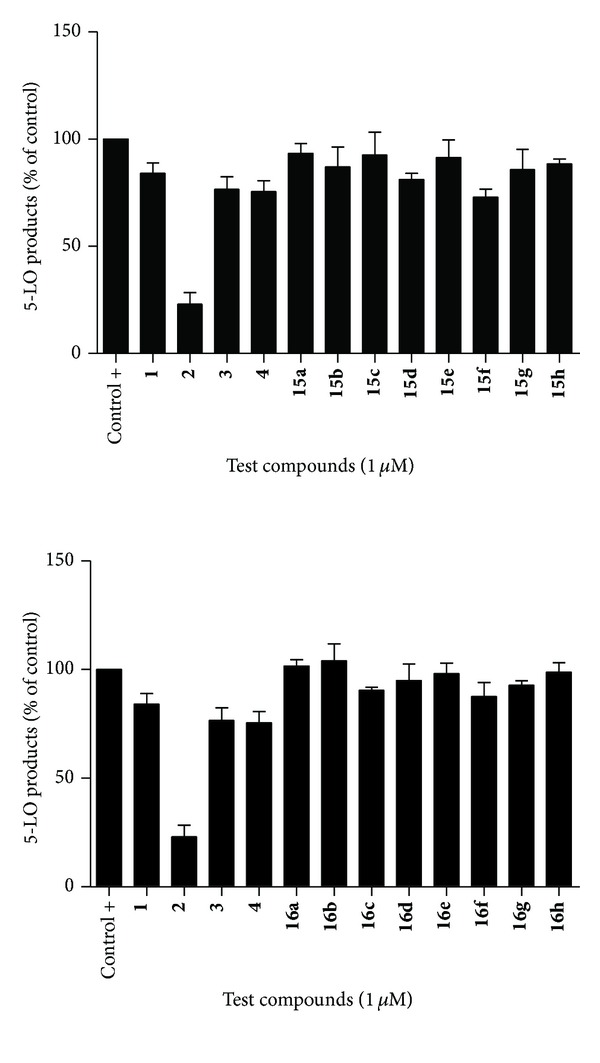
Inhibition of 5-LO products synthesis by caffeic esters (**15a–h**) and amides (**16a–h**) in thapsigargin stimulated human PMNL.

Human PMNL were prepared from peripheral blood as described [26] and were suspended in HBSS containing 1.6 mM CaCl_2_ (10^7^ cells/mL) and preincubated with compounds for 5 min at 37°C in the presence of 1 U/mL of adenosine deaminase (Sigma-Aldrich, Oakville, ON, Canada). Cells were then stimulated for 15 min at 37°C with 1 *μ*M thapsigargin (Sigma-Aldrich) [26]. Reactions were stopped by the addition of 0.5 volume of cold MeOH : CH_3_CN (1 : 1) and 50 ng of PGB_2_ as internal standard and samples were stored at −20°C until processing on octadecyl (C18) columns and RP-HPLC analysis as indicated above. Data are expressed as means ± SEM of 3 independent experiments, each performed in duplicate (Figures 4 and 5).

## 3. Results and Discussion

### 3.1. Strategy

The final target molecules, 1-substituted-1,2,3-triazoles and 1,4-disubstituted-1,2,3-triazoles bearing caffeoyl or cinnamoyl ester or amide moieties, were synthesized in such a way as to permit simple and efficient modifications of triazole substituents. The 1,2,3-triazole linker has been shown to be an excellent hydrolysis-stable bioisostere for the amide bond [33, 34] having proven useful in numerous bioactive and bioconjugate compounds [35, 36]. By reacting the appropriate azide derivatives of cinnamoyl or caffeoyl esters or amides with a library of terminal alkynes under copper(I)-catalyzed Huisgen 1,3-dipolar cycloaddition conditions, it was possible to develop four analogous series of clicked molecules bearing variable aliphatic and aromatic substituents.

The substituents were chosen for their electronic and steric properties: appending progressively larger aliphatic moieties (*R*_1_ = H, *n*-propyl, cyclohexyl) not only modifies the steric bulk of the molecule's triazole terminus but also augments the general lipophilicity of those compounds. The progressive addition of unsaturations to the six-membered ring substituent (*R*_1_ = cyclohexyl, cyclohex-1-enyl, phenyl) modulate flexibility of the side chain while appending *p*-substituents to the aromatic moiety should allow investigation of the effects of additional steric bulk (*R*_1_ = *p*-CH_3_-Ph) as well as the presence of an electron withdrawing H-bond acceptor (*p*-CHO-Ph) and of the common *p*-fluorophenyl group.

These four clicked series allow for the direct comparison of related caffeic and cinnamic derivatives, further confirming that 5-LO inhibitory activity is truly derived from the caffeoyl moiety and not from the appended side chain, as the cinnamoyl moiety is not known to exhibit any notable inhibitory capacity for 5-LO [27, 28]. The synthesis of analogous ester and amide series will give further insight into the importance of the ester linkage for bioactivity. The use of a peptide bond between the active caffeoyl pharmacophore and the clicked side chains in lieu of the usual ester linkage commonly found in caffeoyl derivatives will provide molecules with greater hydrolytic stability which could prove to be excellent starting points for the creating of biologically stable inhibitors.

### 3.2. Chemistry

**Scheme 1:**
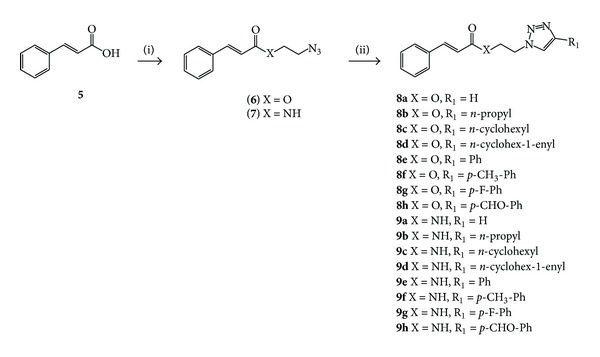
Synthesis of cinnamoyl triazole derivatives** 8a–h **and** 9a–h**. Reagents, conditions, yield: i = SOCl_2_, DMF_cat_, reflux 3 h then N_3_EtOH, pyridine/CH_2_Cl_2_, rt overnight, 72%, or (COCl_2_), DMF_cat_, CH_2_Cl_2_, 0°C 3 h then N_3_EtNH_2_, pyridine/CH_2_Cl_2_, rt overnight, 70%. ii = HCCH, CuI, NEt_3_, DMSO, rt overnight, 68–88%, or R_1_CCH, CuI, NEt_3_, THF, rt overnight, 11–83%.

The cinnamyl azides, 2-azidoethyl cinnamate **6 **and N-(2-azidoethyl) cinnamide **7 **(Scheme 1), were obtained in good yields by condensing cinnamoyl chloride with either 2-azidoethanol or 2-azidoethanamine in pyridine/CH_2_Cl_2_ [37]. Cinnamic acid dissolved in refluxing thionyl chloride with catalytic DMF was the preferred route for the synthesis of acyl chlorides destined for the synthesis of the ester, while the acyl chloride synthesized from cold oxalyl chloride in CH_2_Cl_2_ yielded much cleaner crude reaction mixtures and higher yields in the synthesis of the cinnamyl amide. Monosubstituted cinnamic triazoles **8a **or **9a** (Scheme 1) were obtained by direct reaction of azide precursors **6** or **7 **with acetylene gas, copper (I) iodide, and triethylamine in DMSO to give the expected products in good to excellent yields [38]. 1,4-Disubstituted cinnamic triazoles **8b–h** or **9b–h** (Scheme 1) were obtained via standard anhydrous copper(I)-catalyzed Huisgen 1,3-dipolar cycloaddition conditions by combining the necessary azide precursors **6 **or **7 **and the required terminal alkynes with copper (I) iodide and triethylamine in THF to yield the expected clicked compounds in fair to moderate yields. In general, cinnamide triazoles were obtained in lower yields than the corresponding cinnamates, possibly due to coordination of Cu (I) by the amide nitrogen of cinnamides. Aqueous CuAAC conditions utilizing CuSO_4_ and ascorbic acid in THF/H_2_O did not improve yields and yielded complex reaction mixtures that were difficult to purify.

**Scheme 2:**
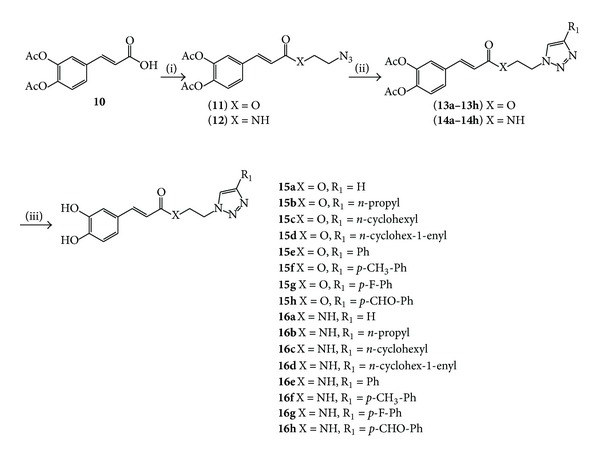
Synthesis of caffeoyl triazole derivatives** 15a–h** and** 16a–h**. Reagents, conditions, yield: i = SOCl_2_, DMF_cat_, reflux 3 h then N_3_EtOH, pyridine/CH_2_Cl_2_, rt overnight, 65%, or (COCl_2_), DMF_cat_, CH_2_Cl_2_, 0°C 3 h then N_3_EtNH_2_, pyridine/CH_2_Cl_2_, rt overnight, 71%. ii = HCCH, CuI, NEt_3_, DMSO, rt overnight, 81%, or R_1_CCH, CuSO_4_-5H_2_O, sodium ascorbate, H_2_O/THF, rt overnight, 43–93%. iii = guanidinium HCl, NEt_3_, MeOH/CH_2_Cl_2_, rt 4h, 28–81%.

Caffeoyl azides, 2-azidoethyl diacetyl caffeate  **11 **and N-(2-azidoethyl) diacetyl caffeamide  **12 **(Scheme 2), were obtained similar to their cinnamyl analogs by condensing diacetylcaffeoyl chloride with either 2-azidoethanol or 2-azidoethanamine in pyridine/CH_2_Cl_2_ [37].

Again, refluxing thionyl chloride with catalytic DMF was used in the synthesis of the caffeate while cold oxalyl chloride in CH_2_Cl_2_ was preferred in the synthesis of the caffeamide. Monosubstituted caffeic triazoles **13a **or **14a** (Scheme 2) were obtained by the same route as their cinnamic analogs by direct reaction of azide precursors **11** or **12 **with acetylene gas, copper (I) iodide, and triethylamine in DMSO to give the expected products in good to excellent yields.

Clicked diacetylcaffeic derivatives **13b–h **and **14b–h **(Scheme 2) were obtained under standard aqueous copper(I)-catalyzed Huisgen 1,3-dipolar cycloaddition conditions by combining the necessary azide precursors **11 **or **12 **and the required terminal alkynes with copper (II) sulfate pentahydrate in a 1 : 1 mixture of THF and water to yield the expected clicked compounds in moderate to good yields. Deacetylation of compounds **13a–h **and **14a–h **to afford caffeic esters **15a–h** and amides **16a–h **(Scheme 2) was performed with guanidinium hydrochloride and triethylamine in MeOH/CH_2_Cl_2_ [39], yielding the expected phenolic compounds in good to excellent yields.

### 3.3. Biology

#### 3.3.1. Radical Scavenging Activity

Radical scavenging activities of analogous cinnamoyl esters **8a–h,** cinnamoyl amides **9a–h**, caffeoyl esters **15a–h**, and caffeoyl amides **16a–h** were assayed using 2,2-diphenyl-1-picrylhydrazyl (DPPH) as a stable radical [25] and are expressed as IC_50_ concentrations in Table 1. Unsurprisingly, cinnamoyl esters **8a–h **and amides **9a–h **(Scheme 1) had no measurable radical scavenging activity up to 200 *μ*M owing to the lack of the redox active catechol moiety present in caffeoyl derivatives, which showed complete quenching of the DPPH stable radical in the 50–75 *μ*M range. This finding supports past investigation into catechol-based antioxidants showing an important link between the number of free hydroxyl groups and radical scavenging activity [40, 41], with cinnamic derivatives possessing no hydroxyl groups.

Caffeate triazoles **15a–h** generally outperformed corresponding caffeamide triazoles **16a–h **(Scheme 2), ascorbic acid, CAPE (**2,** Figure 1), and lead compounds **3** and **4** (Figure 1) in terms of potency (Table 1). That the esters as a whole were slightly more potent radical scavengers than the corresponding amides could indicate a long distance influence of the amide/ester group on the electron accepting ability of the catechol ring, transmitted through the extend *π* system of the conjugated caffeoyl moiety. Similar effects have been noted in DPPH assays with dihydroxybenzamide and dihydroxybenzoic acid, with the benzamide being slightly less potent than the corresponding benzoic acid [42]. That being said, no large differences in radical scavenging capacity were found between triazole esters or amides, with most falling in the IC_50_ range of 8–10 *μ*M and 10–18 *μ*M, respectively. These findings reinforce the notion that while the side chain substituents of caffeoyl derivatives have important influences on compound lipophilicity, steric bulk, and enzyme interactions (which may all be important factors in biological activity of said compounds), radical scavenging ability is influenced mainly by groups having direct influence on the aromatic phenolic moiety [32, 41].

#### 3.3.2. Inhibition of 5-LO Products Synthesis in Whole HEK293 Cells

The inhibitory capacities of synthesized caffeic and cinnamic esters and amides were first evaluated in intact HEK293 cells that were stably transfected with 5-LO and FLAP, thereby possessing all of the necessary cellular machinery required for LTs biosynthesis [28]. Zileuton (**1**) and known caffeic acid-based 5-LO inhibitor CAPE (**2**), whose inhibitory potency was used as a reference, were simultaneously assayed as points of comparison.

As shown in Figure 2, cinnamic esters **8a–h** and amides **9a–h** (Scheme 1) possessed little or no significant 5-LO inhibition at concentrations of 1 *μ*M, where CAPE (**2**) reduced 5-LO products synthesis to roughly 50% of the control value. These results, which are in accordance with past findings, support the notion that 5-LO inhibition in phenylpropanoid-based compounds is strongly dependent on the presence of the catechol moiety found in caffeoyl derivatives and absent in cinnamoyl based-compounds [26–28]. No notable difference in activity was found between corresponding cinnamates and cinnamides.

Along with Zileuton (**1**, 10 *μ*M) and CAPE (**2)**, caffeoyl esters **15a–h** and amides **16a–h** (Scheme 2) were evaluated with caffeic acid-based lead compounds **3** and **4** (Figure 1) for inhibition of 5-LO products synthesis in whole HEK293 cells (Figure 3). It had been previously found that compound **4**, a clicked caffeoyl ester, had excellent inhibitory capacity on HEK293 cells at a concentration of 10 *μ*M [27, 28]. However, as shown in Figure 3, effects on 5-LO were not significantly different than the control at a concentration of 1 *μ*M, where CAPE (**2**) induced roughly 50% inhibition of LTs biosynthesis. Interestingly, though most of the caffeoyl esters and amides had little or no significant effect on LTs biosynthesis at assayed concentrations, esters **15f** and **15g **(Scheme 2) had similar potencies to CAPE (**2**) in HEK293 cell based assays.

Bearing, respectively, a *p*-methylphenyl and *p*-fluorophenyl group tied to the 4-position of the triazole ring, these compounds were more potent than Zileuton (**1**), which had little to no activity at 1 *μ*M and roughly 25% inhibition at 10 *μ*M (not shown). The corresponding amides, **16f** and **16g **(Scheme 2), showed no significant 5-LO inhibitory activity. The finding that analogous caffeic amides are less effective than their related caffeic esters supports findings that CAPE (**2**) suffers important loss of potency when its internal ester linkage is exchanged for an amide bond [26]. As was shown in Table 1, radical scavenging activities of caffeamides are only slightly lower than their related caffeates, suggesting a more complex inhibition mechanism than a simple redox interference with 5-LO nonheme iron. Though compounds **15f** and **15g** are encouraging, no particular structural or electronic property of the appended triazolic moiety seems to clearly induce superior bioactivity in HEK293-based assays (Figure 3).

#### 3.3.3. Inhibition of 5-LO Products Synthesis in Thapsigargin-Stimulated Human PMNL

In order to further probe inhibitory capacity of clicked ester and amide derivatives, tests were undertaken to investigate inhibition of 5-LO in stimulated human PMNL in the presence of 1 *μ*M of the test compounds. 5-LO is highly expressed in PMNL and these cells are important producers of LTB_4_ [28, 32]. As can be seen in Figure 4, cinnamic esters and amides showed no appreciable inhibition capacity for 5-LO in PMNL at 1 *μ*M, once again supporting past findings indicating that the cinnamic moiety has little effect on the LTs synthesis pathway [26–28].

Caffeic esters and amides showed limited activity in PMNL when compared to known caffeic acid-based inhibitor CAPE (**2**) (Figure 4). However, lead compounds **3 **and **4 **(Figure 1), which had never been tested on PMNL-based assays, as well as esters **15d**, **15f**, and **15h **(Scheme 2), were shown to be roughly equipotent with Zileuton (**1**, Figure 1) at 1 *μ*M. The marked difference in inhibitory capacity between CAPE (**2)** and caffeic esters **15a–h **(Scheme 2), which both retain the caffeic ester pharmacophore and differ in structure by thesubstituent on the ester group, highlights the very real importance of the lipophilic moiety distal to the caffeic pharmacophore. Substituting the terminal phenyl group in CAPE (**2**) with a monosubstituted 1H-1,2,3-triazole yields compound **15a** (Scheme 2) and is accompanied by complete loss of inhibition of 5-LO in stimulated PMNL at 1 *μ*M, where CAPE (**2**) induces roughly 75% inhibition. The addition of various lipophilic aliphatic and aromatic groups had little effect on increasing potency of clicked compounds, while exchanging the ester group for an amide group (compounds **16a–h**), which had shown to have important influence on inhibition in previous studies [26], had very little effect on potency.

The markedly lower inhibitory potency of esters **15f **and **15g** (Scheme 2) in the PMNL assays (Figure 5) is found when compared to inhibition tests of these compounds in HEK293 cells (Figure 3). These findings further support the notion that, though a catechol group may be of paramount importance in the design of efficient redox inhibitors, the molecule as a whole plays an important role in assuring efficient enzyme inhibition, where cellular conditions may have important effects on bioavailability and cell penetration of said inhibitors. Though it is suspected and logical to expect caffeic acid-based inhibitors to operate via redox-type mechanism, with X-ray diffraction data of human 5-LO now available, molecular docking studies may give clues to the further more complex inhibitory mechanism, which may explain the varying bioactivities of this class of compounds.

To better determine the potency of the compounds that demonstrated significant inhibitory activities against 5-LO at 1 *μ*M in HEK293 cells, promising inhibitors **15f**, **15g** as well as their corresponding amides **16f** and **16g**, respectively, were selected for further investigation in concentration-response studies in HEK293 cells and in thapsigargin-stimulated human PMNL. The results are summarized in Table 2.

All these compounds inhibited 5-LO products synthesis in a concentration-dependent manner in HEK293 cells and in thapsigargin-stimulated human PMNL and showed prominent inhibitory activities with IC_50_ values ranging from 1.6 to 7.6 *μ*M in HEK293 cells and from 3.1 to 8.8 *μ*M in human PMNL. In HEK293 cells, the clicked caffeic esters bearing a *para*-methylphenyl moiety (**15f**) or a *para*-fluorophenyl moiety (**15g**) were four times more potent than Zileuton (**1**). The corresponding clicked caffeic amides **16f** and **16g** were equipotent to Zileuton (**1**). In thapsigargin-stimulated human PMNL, the same esters (**15f** and **15g**) were equivalent to Zileuton (**1**) while the corresponding amides **16f** and **16g** were less active than Zileuton (**1**) (Table 2).

## 4. Conclusion

In summary, four analogous series of cinnamic and caffeic esters and amides, based on previously discovered lead compound 3, have been synthesised and tested for radical scavenging via DPPH assays and for 5-LO inhibition potential in HEK293 cells as well as in stimulated human PMNL. All caffeic acid-based compounds far outperformed cinnamic analogs in radical scavenging activity though little variation in potency was found through variation of triazole substituents, with slight improvements in activity when comparing related caffeates to caffeamides. No tested cinnamic acid-based compounds were significantly different than control in both 5-LO inhibition assays, while esters **15f** and **15g** were equipotent to known 5-LO inhibitor CAPE (**2**) and more potent than the clinical inhibitor Zileuton **1 **in HEK293 cells. It was found that no tested caffeates or caffeamides had potencies rivalling CAPE (**2) **in stimulated human PMNL, highlighting the clear need for better comprehension of the inhibition mechanism of these compounds in order to extend the use of caffeic acid-based inhibitors to wider, more *in vivo* systems.

## Supplementary Material

Supporting information including synthetic details and spectroscopic analysis of **8c-h**, **9a-h**, **13a**, **13ch**, **14a-h**, **15b-h** and **16a-h**.

## Figures and Tables

**Table 1:** Radical scavenging assay of cinnamoyl esters (**8a–h**) and amides (**9a–h**) and caffeoyl esters (**15a–h**) and amides (**16a–h**).

Compounds	Radical scavenging IC_50_ (*μ*M) [SD]	Compounds	Radical scavenging IC_50_ (*μ*M) [SD]
**8a–h**	>100	**9a–h**	>100
**15a**	9.55 [0.3]	**16a**	15.4 [0.15]
**15b**	9.37 [0.21]	**16b**	12.1 [0.18]
**15c**	9.3 [0.11]	**16c**	9.87 [0.06]
**15d**	8.7 [0.47]	**16d**	11.2 [0.17]
**15e**	15.1 [2.26]	**16e**	10.5 [0.24]
**15f**	9.01 [0.31]	**16f**	13 [0.61]
**15g**	9.4 [0.24]	**16g**	12.01 [0.14]
**15h**	8.85 [0.05]	**16h**	18.1 [1.2]
Zileuton **1**	>100	**3**	12 [0.1]
CAPE **2**	16.5 [4]	**4**	13.4 [0.15]
Ascorbic acid	75.4 [4.1]		

**Table 2:** Determination of IC_50_ values of selected inhibitors.

Compound	HEK293 IC_50_ (*μ*M) [SD]	Human PMNL IC_50_ (*μ*M) [SD]
**1**	8.4 [1.4]	2.7 [0.4]
**15f**	2.1 [0.3]	3.1 [0.2]
**15g**	1.6 [0.2]	3.3 [0.2]
**16f**	5.9 [1.3]	8.2 [0.8]
**16g**	7.6 [1.4]	8.8 [0.7]
