# Supplementary material for: Clicked Cinnamic/Caffeic Esters and Amides as Radical Scavengers and 5-Lipoxygenase Inhibitors
Source: Int J Med Chem. 2014 Feb 18;2014:931756. doi: 10.1155/2014/931756 (PMC4207410; doi:10.1155/2014/931756)
Supplement: Supplementary file 1 — Supporting information including synthetic details and spectroscopic analysis of 8c-h, 9a-h, 13a, 13ch, 14a-h, 15b-h and 16a-h. [file 931756.f1.pdf]

Supporting Information

To accompany

Clicked cinnamic/caffeic esters and amides as radical scavengers and 5-lipoxygenase inhibitors.

Jérémie A. Doiron[a], Benoît Métayer[a], Ryan R. Richard[a], Dany Desjardins[a], Luc H. Boudreau[a,b], Nathalie A. Levesque[a], Jacques Jean-François[a], Samuel J. Poirier[a], Marc E. Surette[a], Mohamed Touaibia[a]*.

[a] Département de chimie et biochimie, Université de Moncton, Moncton, NB, Canada

[b] Centre de Recherche en Rhumatologie et Immunologie et Faculté de Médecine, Université Laval, Québec, Canada QC

*Author to whom correspondence should be addressed;

E-Mail: mohamed.touaibia@umoncton.ca;

Tel.: +1-506-858-4493; Fax: +1-506-858-4541.

Chemistry

All chemicals used were purchased from Aldrich (CA) and used without further purification. Purification of compounds was carried out by silica gel circular chromatography (Chromatotron®, model 7924, Harrison Research) or by flash chromatography. TLC was run on silica gel coated aluminium sheets (SiliaPlate TLC, Silicycle®) with detection by UV light (254 nm, UVS-11, Mineralight® shortwave UV lamp). Melting points were obtained using a MELTEMP® (model 1001D) melting point apparatus. FTIR spectra were recorded on a Nicolet® Impact 400 spectrometer. NMR spectra were recorded on a Bruker® Avance III 400 MHz spectrometer using TMS as an internal standard. High-resolution mass measurements were performed on a Bruker® Doltonics' micrOTOF instrument in positive or negative electrospray.

General procedure I – Monosubstituted triazoles from organic azides and acetylene

To a vigorously stirred solution of the appropriate organic azide (1 mmol, 1 eq.) in 6 mL DMSO is added copper (I) iodide (0.1 mmol, 0.1 eq), after which the reaction vessel is thoroughly flushed with acetylene gas and sealed under balloon pressure. Triethylamine (1.2 mmol, 1.2 eq.) is then added and the mixture is left to react overnight at room temperature. The resulting solution is partitioned between 125 mL of brine and 25 mL ethyl acetate, after which the aqueous phase is extracted three more times with 25 mL ethyl acetate. The organic phase is then washed twice with brine, treated with charcoal, dried over $MgSO_4$ and concentrated. The resulting oil is purified by silica gel circular chromatography (Chromatotron® model 7924, Harrison Research, eluent: $MeOH/CH_2Cl_2$).

General procedure IIA – Anhydrous CuAAC reaction

To a vigorously stirred solution of the appropriate organic azide (0.5 mmol, 1 eq.) and alkyne (0.75 mmol, 1.5 eq.) in 4 mL THF, is added copper (I) iodide (0.025 mmol, 0.05 eq) followed by triethylamine (0.6 mmol, 1.2 eq). The reaction mixture is stirred overnight under argon atmosphere. The resulting solution is partitioned between 30 mL AcOEt and 30 mL H_2O, after

which the aqueous phase is extracted twice more with 30 mL AcOEt. The combined organic fractions are washed twice with saturated ammonium chloride (20 mL), twice with brine, dried over $MgSO_4$ and concentrated. The resulting oil is purified by silica gel circular chromatography (Chromatotron® model 7924, Harrison Research, eluent: AcOEt/Hex or $MeOH/CH_2Cl_2$).

General procedure IIB – Aqueous CuAAC reaction

To a vigorously stirred solution of the appropriate organic azide (0.5 mmol, 1 eq.) and alkyne (0.75 mmol, 1.5 eq.) in 2.5 mL THF is added $CuSO_4 \cdot 5H_2O$ (0.05 mmol, 0.1 eq) dissolved in 2.5 mL H_2O followed by sodium ascorbate (0.05 mmol, 0.1 eq), after which the mixture is left to react overnight. The resulting solution is then diluted to 30 mL with water and extracted three times with AcOEt (20 mL). The organic fractions are then combined, washed twice with water, twice with saturated ammonium chloride, twice with brine, dried over $MgSO_4$ and concentrated. The resulting oil is purified by silica gel circular chromatography (Chromatotron® model 7924, Harrison Research, eluent: AcOEt/Hex or $MeOH/CH_2Cl_2$).

General procedure III – Deacetylation of diacetylcaffeoyl derivatives

The appropriate diacetylcaffeoyl derivative (0.25 mmol, 1 eq) is dissolved in 2 mL anhydrous CH_2Cl_2 under N_2, to which is added 4 mL MeOH. To the resulting stirred solution is added guanidinium hydrochloride (0.81 mmol, 3.25 eq.) followed by triethylamine (2.44 mmol, 9.75 eq). After consumption of the diacetylated precursor (about 2h), the reaction mixture is concentrated and partitioned between 60 mL AcOEt and 30 mL water. The organic phase is then washed again with water, twice with saturated ammonium chloride, twice with brine, treated with charcoal, dried over $MgSO_4$ and concentrated to give the resulting pure caffeoyl derivative as fine powders.

*2-(4-Cyclohexyl-1H-1,2,3-triazol-1-yl)ethyl cinnamate (**8c**)*

Following general procedure IIA with azide **6** and ethynylcyclohexane**,** compound **8c** was obtained as a white solid after silica gel circular chromatography (0 - 25% AcOEt/Hex), yield = 38%. Mp = 119 °C, R_f = 0.37 (50% AcOEt/Hex). ^{1}H NMR (400 MHz, $CDCl_3$, 25 °C), δ (ppm) = 7.71 (d, 1H, J = 16.0 Hz, $=CHC_{ar}$), 7.56 – 7.54 (m, 2H, H_{ar}), 7.43 – 7.40 (m, 3H, H_{ar}), 7.35 (s, 1 H, =CHN), 6.44 (d, 1H, J = 16.0 Hz, =CHCO), 4.68 (t, 2H, J = 5.1 Hz, OCH_2), 4.62 (t, 2H, J = 5.1 Hz, CH_2N), 2.81 – 2.77 (m, 1H, CH-triazole), 2.09 – 2.04 (m, 2H, cyclohexyl), 1.86 – 1.80 (m, 2H, cyclohexyl), 1.49 – 1.36 (m, 4H, cyclohexyl), 1.31 – 1.25 (m, 2H, cyclohexyl); ^{13}C NMR (101 MHz, $CDCl_3$, 25 °C), δ (ppm) = 166.3, 154.0, 146.0, 134.0, 130.7, 129.0, 128.2, 119.9, 116.9, 62.6, 49.0, 35.3, 33.0, 26.1, 26.0. HRMS m/z calc. for $C_{19}H_{23}N_3O_2$ + H^+: 326.1863; detected: 326.1860.

*2-(4-(Cyclohex-1-en-1-yl)-1H-1,2,3-triazol-1-yl)ethyl cinnamate (**8d**)*

Following general procedure IIA with azide **6** and 1-ethynylcyclohex-1-ene**,** compound **8d** was obtained as a white solid after silica gel circular chromatography (0 - 30% AcOEt/Hex), yield = 79%. Mp = 101 - 103 °C, R_f = 0.51 (50% AcOEt/Hex). ^{1}H NMR (400 MHz, $CDCl_3$, 25 °C), δ (ppm) = 7.71 (d, 1H, J = 16.0 Hz, $=CHC_{ar}$), 7.56 – 7.53 (m, 2H, H_{ar}), 7.50 (s, 1H, =CHN), 7.43 – 7.39 (m, 3H, H_{ar}), 6.56 – 6.54 (m, 1 H, CH=C-triazole), 6.43 (d, 1H, J = 16.0 Hz, =CHCO), 4.70 (t, 2H, J = 5.2 Hz, OCH_2), 4.62 (t, 2H, J = 5.2 Hz, CH_2N), 2.42 – 2.40 (m, 2H, cyclohexenyl), 2.25 – 2.21 (m, 2H, cyclohexenyl), 1.82 – 1.76 (m, 2H, cyclohexenyl), 1.72 – 1.65 (m, 2H, cyclohexenyl); ^{13}C NMR (101 MHz, $CDCl_3$, 25 °C), δ (ppm) = 166.4, 149.8, 146.1, 134.0, 130.7, 129.0, 128.3, 127.2, 125.3, 118.8, 116.9, 62.6, 49.1, 26.4, 25.3, 22.5, 22.2. HRMS m/z calc. for $C_{19}H_{21}N_3O_2$ + H^+: 324.1707; detected: 324.1707.

*2-(4-Phenyl-1H-1,2,3-triazol-1-yl)ethyl cinnamate (**8e**)*

Following general procedure IIA with azide **6** and ethynylbenzene**,** compound **8e** was obtained as a white solid after silica gel circular chromatography (0 - 25% AcOEt/Hex), yield = 67%. Mp = 122 - 123 °C, R_f = 0.40 (50% AcOEt/Hex). ^{1}H NMR (400 MHz, $CDCl_3$, 25 °C), δ (ppm) =

7.88 – 7.86 (m, 3H, H_{ar} + =CHN), 7.73 (d, 1H, J = 16.0 Hz, =CHC_{ar}), 7.55 – 7.53 (m, 2H, H_{ar}), 7.47 – 7.35 (m, 6H, H_{ar}), 6.45 (d, 1H, J = 16.0 Hz, =CHCO), 4.78 (t, 2H, J = 5.1 Hz, OCH_2), 4.69 (t, 2H, J = 5.1 Hz, CH_2N); ^{13}C NMR (101 MHz, $CDCl_3$, 25 °C), δ (ppm) = 166.4, 148.1, 146.3, 134.0, 130.7, 130.5, 129.0, 128.9, 128.3, 125.8, 120.2, 116.8, 62.5, 49.3. HRMS m/z calc. for $C_{19}H_{17}N_3O_2 + H^+$: 320.1394; detected: 320.1319.

*2-(4-(p-Tolyl)-1H-1,2,3-triazol-1-yl)ethyl cinnamate (**8f**)*

Following general procedure IIA with azide **6** and 1-ethynyl-4-methylbenzene**,** compound **8f** was obtained as a white solid after silica gel circular chromatography (0 - 25% AcOEt/Hex), yield = 67%. Mp = 139 - 140 °C, R_f = 0.67 (60% AcOEt/Hex). ^1H NMR (400 MHz, $CDCl_3$, 25 °C), δ (ppm) = 7,83 (s, 1H, =CHN), 7.76 - 7.70 (m, 3 H, H_{ar} + =CHC_{ar}), 7.55 – 7.53 (m, 2H, H_{ar}), 7.42 – 7.41 (m, 3H, H_{ar}), 7.26 (d, 2H, J = 7.9 Hz, H_{ar}), 6.45 (d, 1H, J = 16,0 Hz, =CHCO), 4.76 (t, 2H, J = 4.9 Hz, OCH_2), 4.68 (t, 2H, J = 4.9 Hz, CH_2N), 2.40 (s, 3H, CH_3); ^{13}C NMR (101 MHz, $CDCl_3$, 25 °C), δ (ppm) = 166.4, 148.2, 146.2, 138.1, 134.0, 130.7, 129.6, 129.0, 128.3, 127.6, 125.7, 119.8, 116.8, 62.6, 49.3, 21.3. HRMS m/z calc. for $C_{20}H_{19}N_3O_2 + H^+$: 334.1550; detected: 334.1549.

*2-(4-(4-Fluorophenyl)-1H-1,2,3-triazol-1-yl)ethyl cinnamate (**8g**)*

Following general procedure IIA with azide **6** and 1-ethynyl-4-fluorobenzene**,** compound **8g** was obtained as a white solid after silica gel circular chromatography (0 - 25% AcOEt/Hex), yield = 83%. Mp = 134 - 135 °C, R_f = 0.51 (50% AcOEt/Hex). ^1H NMR (400 MHz, $CDCl_3$, 25 °C), δ (ppm) = 7.85 – 7.81 (m, 3H, H_{ar} + =CHN), 7.73 (d, 1H, J = 16.0 Hz, =CHC_{ar}), 7.54 – 7.52 (m, 2H, H_{ar}), 7.42 – 7.41 (m, 3H, H_{ar}), 7.14 (t, 2H, J = 8.7 Hz, H_{ar}), 6.45 (d, 1H, J = 16.0 Hz, =CHCO), 4.77 (t, 2H, J = 5.0 Hz, OCH_2), 4.69 (t, 2H, J = 5.0 Hz, CH_2N); ^{13}C NMR (101 MHz, $CDCl_3$, 25 °C), δ (ppm) = 166.4, 164.0, 161.5, 147.3, 146.3, 134.0, 130.8, 129.0, 128.3, 127.6, 127.5, 126.7, 126.7, 119.9, 116.7, 116.0, 115.8, 62.5, 49.3. HRMS m/z calc. for $C_{19}H_{16}FN_3O_2$ + H^+: 338.1299; detected: 338.1294.

*2-(4-(4-Formylphenyl)-1H-1,2,3-triazol-1-yl)ethyl cinnamate (**8h**)*

Following general procedure IIA with azide **6** and 4-ethynylbenzaldehyde**,** compound **8h** was obtained as a white solid after silica gel circular chromatography (0 - 50% AcOEt/Hex), yield = 79%. Mp = 127 - 128 °C, R_f = 0.39 (50% AcOEt/Hex). ^{1}H NMR (400 MHz, $CDCl_3$, 25 °C), δ (ppm) = 10.05 (s, 1H, CHO), 8.05 (d, 2H, J = 8.1 Hz, H_{ar}), 7.98 (d, 2H, J = 8.1 Hz, H_{ar}), 7.73 (d, 1H, J = 16.0 Hz, =CHC_{ar}), 7.55 – 7.53 (m, 2H, H_{ar}), 7.45 – 7.39 (m, 3H, H_{ar}), 6.45 (d, 1H, J = 16.0 Hz, =CHCO), 4.81 (t, 2H, J = 5.1 Hz, OCH_2), 4.71 (t, 2H, J = 5.1 Hz, CH_2N); ^{13}C NMR (101 MHz, $CDCl_3$, 25 °C), δ (ppm) = 166.3, 146.8, 146.4, 136.2, 135.9, 133.9, 130.8, 130.4, 129.0, 128.3, 126.1, 121.3, 116.6, 62.4, 49.5. HRMS m/z calc. for $C_{20}H_{17}N_3O_3$: 348.1343; detected : 348.1339.

*N-(2-(1H-1,2,3-Triazol-1-yl)ethyl)cinnamamide (**9a**)*

Following general procedure I with azide **7,** compound **9a** was obtained as a beige powder after silica gel circular chromatography (0 – 1.5% MeOH/CH_2Cl_2), yield = 68%. Mp = 140 – 141 °C, R_f = 0.25 (7.5% MeOH/CH_2Cl_2). ^{1}H NMR (400 MHz, $CDCl_3$, 25 °C), δ (ppm) = 7.70 (s, 1H, =CHN), 7.65 (d, 1H, J = 15.7 Hz, =CHC_{ar}), 7.61 (s, 1H, =CHN), 7.50 (m, 2H, H_{ar}), 7.37 (m, 3H, H_{ar}), 6.67 (br s, 1H, NH), 6.45 (d, 1H, J = 15.7 Hz, =CHCO), 4.63 (t, 2H, J = 5.7 Hz, CH_2-triazole), 3.95 (q, 2H, J = 5.7 Hz, NH**C**$\mathbf{H_2}$). ^{13}C NMR (101 MHz, $CDCl_3$, 25 °C), δ (ppm) = 166.51, 141.70, 134.56, 133.82, 129.92, 128.86, 127.89, 124.64, 120.00, 49.45, 39.58. HRMS m/z calc. for $C_{13}H_{14}N_4O + (H^+)$: 243.1240; detected : 243.1244.

*N-(2-(4-Propyl-1H-1,2,3-triazol-1-yl)ethyl)cinnamamide. (**9b**)*

Following general procedure IIA with azide **7** and 1-pentyne**,** compound **9b** was obtained as white crystals after silica gel circular chromatography (0 - 6% MeOH/CH_2Cl_2), yield = 39%. Mp = 156 - 157 °C, R_f = 0.53 (10% MeOH/CH_2Cl_2). ^{1}H NMR (400 MHz, $CDCl_3$, 25 °C), δ (ppm) = 7.65 (d, 1H, J = 15.6 Hz, =CHC_{ar}), 7.54 – 7.49 (m, 2H, H_{ar}), 7.42 – 7.30 (m, 4H, H_{ar} + =CHN), 6.15 (br s, 1H, NH), 6.43 (d, 1H, J = 15.6 Hz, =CHCO), 4.54 (t, 2H, J = 5.6 Hz, CH_2-triazole), 3.94 (q, 2H, J = 5.6 Hz, CH_2NH), 2.69 (t, 2H, J = 7.6 Hz, $\mathbf{CH_2}CH_2CH_3$), 1.65 – 1.75 (m, 2H,

$CH_2\mathbf{CH_2}CH_3$), 0.98 (t, 2H, J = 7.4 Hz, CH_3); ^{13}C NMR (101 MHz, $CDCl_3$, 25 °C), δ (ppm) = 166.38, 141.63, 134.61, 129.87, 128.85, 127.87, 120.08, 49.38, 39.47, 27.62, 22.66, 13.78. HRMS m/z calc. for $C_{16}H_{20}N_4O + H^+$: 285.1710; detected: 285.1702.

*N-(2-(4-Cyclohexyl-1H-1,2,3-triazol-1-yl)ethyl)cinnamamide (**9c**)*

Following general procedure IIA with azide **7** and ethynylcyclohexane**,** compound **9c** was obtained as white crystals after silica gel circular chromatography (0 - 4% $MeOH/CH_2Cl_2$), yield = 18%. Mp = 159 - 161 °C, R_f = 0.40 (6% $MeOH/CH_2Cl_2$). ^1H NMR (400 MHz, $CDCl_3$, 25 °C), δ (ppm) = 7.65 (d, 1H, J = 15.6 Hz, $=CHC_{ar}$), 7.52 - 7.50 (m, 2H, H_{ar}), 7.41 – 7.37 (m, 4H, H_{ar} + =CHN), 6.69 (br s, 1H, NH), 6.46 (d, 1H, J = 15.6 Hz, =CHCO), 4.50 (t, 2H, J = 5.6 Hz, CH_2-triazole), 3.95 (q, 2H, J = 5.6 Hz, CH_2NH), 2.72 – 2.60 (m, 1H, CH-triazole), 1.81 – 1.71 (m, 4H, cyclohexyl), 1.38 – 1.42 (m, 4H, cyclohexyl), 1.27 – 1.24 (m, 2H, cyclohexyl); ^{13}C NMR (101 MHz, $CDCl_3$, 25 °C), δ (ppm) = 166.43, 141.53, 134.65, 129.84, 128.84, 127.88, 120.23, 49.52, 39.42, 35.26, 32.92, 26.09, 25.98. HRMS m/z calc. for $C_{19}H_{24}N_4O + H^+$: 325.2023; detected: 325.2014.

*N-(2-(4-(Cyclohex-1-en-1-yl)-1H-1,2,3-triazol-1-yl)ethyl)cinnamamide (**9d**)*

Following general procedure IIA with azide **7** and 1-ethynylcyclohex-1-ene**,** compound **9d** was obtained as a white solid after silica gel circular chromatography (0 - 4% $MeOH/CH_2Cl_2$), yield = 24%. Mp = 170 - 171 °C, R_f = 0.44 (6% $MeOH/CH_2Cl_2$). ^1H NMR (400 MHz, acetone-d_6, 25 °C), δ (ppm) = 7.90 (s, 1H, =CHN), 7.59 – 7.52 (m, 4H, NH + $=CHC_{ar}$ + H_{ar}), 7.42 – 7.36 (m, 3H, H_{ar}), 6.68 (d, 1H, J = 15.6 Hz, =CHCO), 6.48 - 6.45 (m, 1H, CH=C-triazole), 4.56 (t, 2H, J = 5.6 Hz, CH_2-triazole), 3.82 (q, 2H, J = 5.6 Hz, CH_2NH), 2.40 – 2.34 (m, 2H, cyclohexenyl), 2.20 – 2.14 (m, 2H, cyclohexenyl), 1.77 – 1.70 (m, 2H, cyclohexenyl), 1.70 – 1.61 (m, 2H, cyclohexenyl); ^{13}C NMR (101 MHz, acetone-d_6, 25 °C), δ (ppm) = 165.49, 148.61, 139.73, 135.22, 129.43, 128.82, 127.93, 123.26, 121.51, 119.47, 49.14, 39.48, 25.98, 24.88, 22.34, 22.16. HRMS m/z calc. for $C_{19}H_{22}N_4O + H^+$: 323.1866; detected: 323.1857.

*N-(2-(4-Phenyl-1H-1,2,3-triazol-1-yl)ethyl)cinnamamide (**9e**)*

Following general procedure IIA with azide **7** and ethynylbenzene**,** compound **9e** was obtained as a white solid after silica gel circular chromatography (0 - 7% MeOH/CH_2Cl_2), yield = 16%. Mp = 171 - 173 °C, R_f = 0.40 (36% MeOH/CH_2Cl_2). ^{1}H NMR (400 MHz, DMSO-d_6, 25 ^{o}C), δ (ppm) = 8.60 (s, 1H, =CHN), 8.30 (m, 1H, NH), 7.87 – 7.84 (m, 2H, H_{ar}), 7.60 – 7.25 (m, 9H, =CHC_{ar} + H_{ar}), 6.60 (d, 1H, J = 15.6 Hz, =CHCO), 4.54 (t, 2H, J = 6.0 Hz, CH_2-triazole), 3.70 (q, 2H, J = 6.0 Hz, CH_2NH). ^{13}C NMR (101 MHz, DMSO-d_6, 25 ^{o}C), δ (ppm) = 165.88, 146.75, 139.56, 135.20, 131.31, 130.01, 129.39, 129.35, 128.26, 128.03, 125.57, 122.17, 49.62, 39.45. HRMS m/z calc. for $C_{19}H_{18}N_4O$ + H^+: 319.1553; detected: 319.1553.

*N-(2-(4-(p-Tolyl)-1H-1,2,3-triazol-1-yl)ethyl)cinnamamide (**9f**)*

Following general procedure IIA with azide **7** and 1-ethynyl-4-methylbenzene**,** compound **9f** was obtained as a white solid after silica gel circular chromatography (0 - 3% MeOH/CH_2Cl_2), yield = 11%. Mp = 208 °C, R_f = 0.32 (6% MeOH/CH_2Cl_2). ^{1}H NMR (400 MHz, DMSO-d_6, 25 ^{o}C), δ (ppm) = 8.53 (s, 1H, =CHN), 8.33 (t, 1H, J = 5.7 Hz, NH), 7.73 (d, 2H, J = 8.0 Hz, H_{ar}), 7.60 – 7.30 (m, 6H, H_{ar} + =CHC_{ar}), 7.25 (d, 2H, J = 8.0 Hz, H_{ar}), 6.60 (d, 1H, J = 16.0 Hz, =CHCO), 4.52 (t, 2H, J = 5.7 Hz, CH_2-triazole), 3.69 (q, 2H, J = 5.7 Hz, CH_2NH), 2.32 (s, 3H, CH_3); ^{13}C NMR (101 MHz, DMSO-d_6, 25 ^{o}C), δ (ppm) = 165.86, 146.81, 139.54, 137.53, 135.20, 130.01, 129.90, 129.40, 128.54, 128.03, 125.51, 122.16, 121.74, 49.57, 39.45, 21.29. HRMS m/z calc. for $C_{20}H_{20}N_4O$ + H^+: 333.1710; detected: 333.1700.

*N-(2-(4-(4-Fluorophenyl)-1H-1,2,3-triazol-1-yl)ethyl)cinnamamide (**9g**)*

Following general procedure IIA with azide **7** and 1-ethynyl-4-fluorobenzene**,** compound **9g** was obtained as a white solid after silica gel circular chromatography (0 - 6% MeOH/CH_2Cl_2), yield = 41%. Mp = 193 - 196 °C, R_f = 0.52 (10% MeOH/CH_2Cl_2). ^{1}H NMR (400 MHz, acetone-d_6, 25 ^{o}C), δ (ppm) = 8.39 (s, 1H, =CHN), 7.96 – 7.92 (m, 2H, H_{ar}), 7.60 – 7.55 (m, 4H, NH + =CHC_{ar} + H_{ar}), 7.43 – 7.37 (m, 3H, H_{ar}), 7.24 – 7.19 (m, 2H, H_{ar}), 6.68 (d, 1H, J = 16.0 Hz, =CHCO), 4.66 (t, 2H, J = 5.9 Hz, CH_2-triazole), 3.88 (q, 2H, J = 5.9 Hz, CH_2NH). ^{13}C NMR (101 MHz,

acetone-d_6, 25 ^{o}C), δ (ppm) = 165.54, 163.55, 161.12, 146.01, 139.79, 135.20, 129.45, 128.83, 127.97, 127.93, 127.62, 127.25, 127.17, 121.46, 120.89, 115.64, 115.42, 49.46, 39.50. HRMS m/z calc. for $C_{19}H_{17}FN_4O + H^+$: 337.1459; detected: 337.1450.

*N-(2-(4-(4-Formylphenyl)-1H-1,2,3-triazol-1-yl)ethyl)cinnamamide (**9h**)*

Following general procedure IIA with azide **7** and 4-ethynylbenzaldehyde**,** compound **9h** was obtained as a white solid after silica gel circular chromatography (0 - 4% $MeOH/CH_2Cl_2$), yield = 21%. Mp = 200 - 201 °C, R_f = 0.42 (6% $MeOH/CH_2Cl_2$). ^{1}H NMR (400 MHz, DMSO-d_6, 25 ^{o}C), δ (ppm) = 10.01 (s, 1H, CHO), 8.81 (s, 1H, =CHN), 8.35 (t, 1H, J = 5.6 Hz, NH), 8.08 (d, 2H, J = 8.0 Hz, H_{ar}), 7.79 (d, 2H, J = 8.0 Hz, H_{ar}), 7.60 – 7.30 (m, 6H, =CHC_{ar} + H_{ar}), 6.59 (d, 1H, J = 15.6 Hz, =CHCO), 4.57 (t, 2H, J = 5.7 Hz, CH_2-triazole), 3.71 (q, 2H, J = 5.6 Hz, CH_2NH). ^{13}C NMR (101 MHz, DMSO-d_6, 25 ^{o}C), δ (ppm) = 192.98, 165.89, 145.67, 139.58, 136.93, 135.78, 135.18, 130.79, 130.02, 129.40, 128.03, 125.95, 123.81, 122.13, 49.81, 39.42. HRMS m/z calc. for $C_{20}H_{18}N_4O_2 + H^+$: 347.1503; detected: 347.1497.

*(E)-4-(3-(2-(1H-1,2,3-Triazol-1-yl)ethoxy)-3-oxoprop-1-en-1-yl)-1,2-phenylene diacetate (**13a**)*

Following general procedure I with azide **11,** compound **13a** was obtained as a white powder after silica gel circular chromatography (1% $MeOH/CH_2Cl_2$), yield = 81%. Mp = 123 – 125 °C, R_f = 0.43 (4% $MeOH/CH_2Cl_2$). ^{1}H NMR (400 MHz, $CDCl_3$, 25 °C), δ (ppm) = 7.76 (s, 1H, =CHN), 7.67 (s, 1H, =CHN), 7.63 (d, 1H, J = 16.0 Hz, CHC_{ar}), 7.42 (dd, 1H, J = 8.4 Hz, 1.9 Hz, H_{ar}), 7.38 (d, 1H, J = 1.8 Hz, H_{ar}), 7.25 (d, 1H, J = 8.3 Hz, H_{ar}), 6.36 (d, 1H, J = 16.0 Hz, =CHCO), 4.76 (t, 2H, J = 5.0 Hz, OCH_2), 4.64 (t, 2H, J = 5.2 Hz, CH_2N), 2.33 (s, 3H, CH_3COO), 2.32 (s, 3H, CH_3COO). ^{13}C NMR (101 MHz, $CDCl_3$, 25 °C), δ (ppm) = 168.08, 167.96, 165.88, 144.23, 143.82, 142.50, 134.14, 132.81, 126.62, 124.04, 122.87, 117.93, 62.65, 48.99, 20.67, 20.63. HRMS m/z calc. for $C_{17}H_{17}N_3O_6$ + (H^+) : 360.1190; detected : 360.1184.

*(E)-4-(3-(2-(4-Cyclohexyl-1H-1,2,3-triazol-1-yl)ethoxy)-3-oxoprop-1-en-1-yl)-1,2-phenylene diacetate (**13c**)*

Following general procedure IIB with azide **11** and ethynylcyclohexane**,** compound **13c** was obtained as a white solid after silica gel circular chromatography (0 – 2% MeOH/CH_2Cl_2), yield = 78%. Mp = 121 - 124 °C, R_f = 0.31 (2% MeOH/CH_2Cl_2). ^{1}H NMR (400 MHz, $CDCl_3$, 25 °C), δ (ppm) = 7.64 (d, 1H, J = 16.0 Hz, =CHC_{ar}), 7.42 (dd, 1H, J = 8.4 Hz, 2.0 Hz, H_{ar}), 7.38 (d, 1H, J = 2.0 Hz, H_{ar}), 7.33 (s, 1H, =CHN), 7.26 (d, 1H, J = 8.4 Hz, H_{ar}), 6.38 (d, 1H, J = 16,0 Hz, =CHCO), 4.67 (t, 2H, J = 5.0 Hz, OCH_2), 4.61 (t, 2H, J = 5.0 Hz, CH_2N), 2.81 – 2.77 (m, 1H, CH-triazole), 2.33 (s, 3H, CH_3COO), 2.32 (s, 3H, CH_3COO), 2.09 – 2.04 (m, 2H, cyclohexyl), 1.87 – 1.81 (m, 4H, cyclohexyl), 1.49 – 1.36 (m, 4H, cyclohexyl); ^{13}C NMR (101 MHz, $CDCl_3$, 25 °C), δ (ppm) = 168.1, 168.0, 165.9, 154.1, 144.1, 143.8, 142.5, 132.9, 126.6, 124.0, 122.9, 119.8, 118.1, 62.8, 53.4, 48.9, 35.3, 33.0, 26.1, 26.0, 20.7, 20.6. HRMS m/z calc. for $C_{23}H_{27}N_3O_6$ + H^+: 442.1973; detected: 442.1964.

*(E)-4-(3-(2-(4-(Cyclohex-1-en-1-yl)-1H-1,2,3-triazol-1-yl)ethoxy)-3-oxoprop-1-en-1-yl)-1,2-phenylene diacetate (**13d**)*

Following general procedure IIB with azide **11** and 1-ethynyl-1-cyclohexene**,** compound **13d** was obtained as a white solid after silica gel circular chromatography (0 – 0.5% MeOH/CH_2Cl_2), yield = 67%. Mp = 118 - 120 °C, R_f = 0.40 (4% MeOH/CH_2Cl_2). ^{1}H NMR (400 MHz, $CDCl_3$, 25 °C), δ (ppm) = 7.64 (d, 1H, J = 16.0 Hz, =CHC_{ar}), 7.48 (s, 1H, =CHN), 7.42 (dd, 1H, J = 8.4 Hz, 1.7 Hz, H_{ar}), 7.38 (d, 1H, J = 1.7 Hz, H_{ar}), 7.26 (d, 1H, J = 8.4 Hz, H_{ar}), 6.56 – 6.54 (m, 1H, CH=C-triazole), 6.37 (d, 1H, J = 16.0 Hz, =CHCO), 4.69 (t, 2H, J = 5.1 Hz, OCH_2), 4.62 (t, 2H, J = 5.1 Hz, NCH_2), 2.42 – 2.40 (m, 2H, cyclohexenyl), 2.34 (s, 3H, CH_3COO), 2.33 (s, 3H, CH_3COO), 2.25 – 2.21 (m, 2H, cyclohexenyl), 1.82 – 1.76 (m, 2H, cyclohexenyl), 1.72 – 1.65 (m, 2H, cyclohexenyl); ^{13}C NMR (101 MHz, $CDCl_3$, 25 °C), δ (ppm) = 168.08, 167.97, 165.95, 149.95, 144.15, 143.79, 142.49, 132.88, 127.15, 126.63, 125.35, 124.04, 122.86, 118.78, 118.06, 62.73, 49.02, 26.40, 25.27, 22.44, 22.19, 20.67, 20.63. HRMS m/z calc. for $C_{23}H_{25}N_3O_6$ + H^+: 440.1816; detected: 440.1817.

*(E)-4-(3-oxo-3-(2-(4-Phenyl-1H-1,2,3-triazol-1-yl)ethoxy)prop-1-en-1-yl)-1,2-phenylene diacetate (**13e**)*

Following general procedure IIB with azide **11** and ethynylbenzene**,** compound **13e** was obtained as a white solid after silica gel circular chromatography (0 – 40% AcOEt/Hex), yield = 46%. Mp = 158 - 160 °C, R_f = 0.41 (60% AcOEt/Hex). ^{1}H NMR (400 MHz, $CDCl_3$, 25 °C), δ (ppm) = 7.88 – 7.85 (m, 3H, H_{ar} + =CHN), 7.65 (d, 1H, J = 16.0 Hz, =CHC_{ar}), 7.47 – 7.34 (m, 5H, H_{ar}), 7.24 (d, 1H, J = 8.4, H_{ar}), 6.38 (d, 1H, J = 16.0 Hz, =CHCO), 4.76 (t, 2H, J = 5.0 Hz, OCH_2), 4.68 (t, 2H, J = 5.0 Hz, CH_2N), 2.33 (s, 3 H, CH_3COO). 2.32 (s, 3 H, CH_3COO); ^{13}C NMR (101 MHz, $CDCl_3$, 25 °C), δ (ppm) = 168.1, 168.0, 165.9, 148.1, 144.3, 143.8, 142.5, 132.8, 130.4, 128.9, 128.3, 126.7, 125.8, 124.0, 122.9, 120.2, 118.0, 62.7, 49.3, 20.7, 20.6. HRMS m/z calc. for $C_{23}H_{21}N_3O_6 + H^+$: 436.1503; detected: 436.1499.

*(E)-4-(3-oxo-3-(2-(4-(p-Tolyl)-1H-1,2,3-triazol-1-yl)ethoxy)prop-1-en-1-yl)-1,2-phenylene diacetate (**13f**)*

Following general procedure IIB with azide **11** and 1-ethynyl-4-methylebenzene**,** compound **13f** was obtained as a white solid after silica gel circular chromatography (0.4% $MeOH/CH_2Cl_2$), yield = 78%. Mp = 156 - 158 °C, R_f = 0.47 (3% $MeOH/CH_2Cl_2$). ^{1}H NMR (400 MHz, $CDCl_3$, 25 °C), δ (ppm) = 7.82 (s, 1H, =CHN), 7.75 (d, 2H, J = 8.0 Hz, H_{ar}), 7.65 (d, 1H, J = 16.0 Hz, =CHC_{ar}), 7.41 (dd, 1H, J = 8.4 Hz; 1.7 Hz, H_{ar}), 7.37 (d, 1H, J = 1.8 Hz, H_{ar}), 7.28-7.23 (m, 3H, H_{ar}), 6.38 (d, 1H, J = 16.0 Hz, =CHCO), 4.75 (t, 2H, J = 5.0 Hz, OCH_2), 4.67 (t, 2H, J = 5.0 Hz, CH_2N), 2.34 (s, 3H, CH_3C_{ar}), 2.33 (s, 3H, CH_3COO), 2.32 (s, 3H, CH_3COO). ^{13}C NMR (101 MHz, $CDCl_3$, 25 °C), δ (ppm) = 168.07, 167.95, 165.95, 148.18, 144.74, 143.81, 142.49, 138.13, 132.84, 129.56, 127.61, 126.66, 125.70, 124.02, 122.87, 119.81, 117.99, 62.68, 49.21, 21.30, 20.67, 20.62. HRMS m/z calc. for $C_{24}H_{23}N_3O_6 + H^+$: 450.1660; detected: 450.1660.

*(E)-4-(3-(2-(4-(4-Fluorophenyl)-1H-1,2,3-triazol-1-yl)ethoxy)-3-oxoprop-1-en-1-yl)-1,2-phenylene diacetate (**13g**)*

Following general procedure IIB with azide **11** and 1-ethynyl-4-fluorobenzene**,** compound **13g** was obtained as a white solid after silica gel circular chromatography (0 - 1% $MeOH/CH_2Cl_2$),

yield = 93%. Mp = 138 °C, R_f = 0.41 (2% MeOH/CH_2Cl_2). ^{1}H NMR (400 MHz, $CDCl_3$, 25 °C), δ (ppm) = 7.85 – 7.82 (m, 3H, H_{ar} + =CHN), 7.65 (d, 1H, J = 16.0 Hz, =CHC_{ar}), 7.41 (dd, 1H, J = 8.4 Hz, J = 1.8 Hz, H_{ar}), 7.37 (d, 1H, J = 1.8 Hz, H_{ar}), 7.24 (d, 1H, J = 8.4 Hz, H_{ar}), 7.14 (m, 2H, H_{ar}), 6.38 (d, 1H, J = 16.0 Hz, =CHCO), 4.76 (t, 2H, J = 4.9 Hz, OCH_2), 4.68 (t, 2H, J = 4.9 Hz, CH_2N), 2.33 (s, 3H, CH_3COO), 2.32 (s, 3H, CH_3COO); ^{13}C NMR (101 MHz, $CDCl_3$, 25 °C), δ (ppm) = 168.1, 168.0, 165.9, 164.0, 161.5, 147.3, 144.3, 143.8, 142.5, 132.8, 127.6, 127.5, 126.7, 126.6, 124.1, 122.9, 119.9, 117.9, 116.0, 115.8, 62.6, 49.3, 20.7, 20.6. HRMS m/z calc. for $C_{23}H_{20}FN_3O_6 + H^+$: 454.1409; detected: 454.1405.

*(E)-4-(3-(2-(4-(4-Formylphenyl)-1H-1,2,3-triazol-1-yl)ethoxy)-3-oxoprop-1-en-1-yl)-1,2-phenylene diacetate (**13h**)*

Following general procedure IIB with azide **11** and 4-ethynylbenzaldehyde**,** compound **13h** was obtained as a white solid after silica gel circular chromatography (0 - 1% MeOH/CH_2Cl_2), yield = 82%. Mp = 149 - 150 °C, R_f = 0.31 (2% MeOH/CH_2Cl_2). ^{1}H NMR (400 MHz, $CDCl_3$, 25 °C), δ (ppm) = 10.04 (s, 1H, CHO), 8.04 (d, 2H, J = 8.4 Hz, H_{ar}), 7.99 – 7.95 (m, 3 H, H_{ar} + =CHN), 7.64 (d, 1H, J = 16.0 Hz, =CHC_{ar}), 7.42 – 7.46 (m, 2H, H_{ar}), 7.24 (dd, 1 H, J = 8,4 Hz, J = 1,8 Hz, H_{ar}), 6.38 (d, 1H, J = 15.9 Hz, =CHCO), 4.79 (t, 2H, J = 4.9 Hz, OCH_2), 4.70 (t, 2H, J = 4.9 Hz, CH_2N), 2.33 (s, 3H, CH_3COO), 2.32 (s, 3H, CH_3COO); ^{13}C NMR (101 MHz, $CDCl_3$, 25 °C), δ (ppm) = 191.7, 168.1, 168.0, 165.9, 146.8, 144.4, 144.4, 143.9, 142.5, 136.2, 135.9, 132.7, 130.4, 126.6, 126.1, 124.1, 122.9, 121.4, 121.4, 117.8, 62.5, 49.4, 20.7, 20.6. HRMS m/z calc. for $C_{24}H_{21}N_3O_7 + H^+$: 464.1452; detected: 464.1447.

*(E)-4-(3-((2-(1H-1,2,3-Triazol-1-yl)ethyl)amino)-3-oxoprop-1-en-1-yl)-1,2-phenylene diacetate (**14a**)*

Following general procedure I with azide **12,** compound **14a** was obtained as a white powder after silica gel circular chromatography (0.5 – 2% MeOH/CH_2Cl_2), yield = 76%. Mp = 174 – 176 °C, R_f = 0.30 (5% MeOH/CH_2Cl_2). ^{1}H NMR (400 MHz, $CDCl_3$, 25 °C), δ (ppm) = 7.70 (s, 1H, =CHN), 7.60 (s, 1H, =CHN), 7.56 (d, 1H, J = 15.7 Hz, =CHC_{ar}), 7.35 (m, 2H, H_{ar}), 7.20 (d, 1H, J

= 8.5 Hz, H_{ar}), 6.70 (br s, 1H, NH), 6.36 (d, 1H, J = 15.7 Hz, =CHCO), 4.60 (t, 2H, J = 5.7 Hz, CH_2-triazole), 3.91 (q, 2H, J = 5.7 Hz, NHC**H**$_2$), 2.32 (s, 3H, CH_3), 2.31 (s, 3H, CH_3). ^{13}C NMR (101 MHz, $CDCl_3$, 25 °C), δ (ppm) = 168.18, 168.14, 166.03, 143.09, 142.36, 139.73, 133.58, 126.30, 124.65, 123.86, 12243, 121.27, 49.36, 39.58, 20.67, 20.64. HRMS m/z calc. for $C_{17}H_{18}N_4O_5$ + (H^+) : 359.1350; detected : 359.1347.

*(E)-4-(3-oxo-3-((2-(4-Propyl-1H-1,2,3-triazol-1-yl)ethyl)amino)prop-1-en-1-yl)-1,2-phenylene diacetate (**14b**)*

Following general procedure IIB with azide **12** and 1-pentyne**,** compound **14b** was obtained as a white solid after silica gel circular chromatography (0.5 – 1.5% MeOH/CH_2Cl_2), yield = 62%. Mp = 134 – 136 °C, R_f = 0.58 (7.5% MeOH/CH_2Cl_2). ^{1}H NMR (400 MHz, $CDCl_3$, 25 °C), δ (ppm) = 7.60 (d, 1H, J = 15.7 Hz, =CHC_{ar}), 7.37 (dd, 1H, J = 8.4 Hz, 1.7 Hz, H_{ar}), 7.33 (d, 1H, J = 1.7 Hz, H_{ar}), 7.31 (s, 1H, =CHN), 7.21 (d, 1H, J = 8.4 Hz, H_{ar}), 6.61 (br s, 1H, NH), 6.36 (d, 1H, J = 15.7 Hz, =CHCO), 4.52 (t, 2H, J = 5.9 Hz, CH_2-triazole), 3.91 (q, 2H, J = 5.9 Hz, NHC**H**$_2$), 2.69 (t, 2H, J = 7.6 Hz, C**H**$_2CH_2CH_3$), 2.32 (s, 3H, CH_3COO), 2.31 (s, 3H, CH_3COO), 1.70 (m, 2H, CH_2C**H**$_2CH_3$), 0.97 (t, 3H, J = 7.3 Hz, CH_3). ^{13}C NMR (101 MHz, $CDCl_3$, 25 °C), δ (ppm) = 168.12, 168.08, 165.91, 148.33, 143.11, 142.37, 139.73, 133.60, 126.25, 123.85, 122.45, 121.82, 121.29, 49.29, 39.50, 27.60, 22.66, 20.66, 20.62, 13.77**.** HRMS m/z calc. for $C_{20}H_{24}N_4O_5$ + (H^+) : 401.1819; detected : 401.1826

*(E)-4-(3-((2-(4-Cyclohexyl-1H-1,2,3-triazol-1-yl)ethyl)amino)-3-oxoprop-1-en-1-yl)-1,2-phenylene diacetate (**14c**)*

Following general procedure IIB with azide **12** and ethynylcyclohexane**,** compound **14c** was obtained as a white solid after silica gel circular chromatography (0 – 1.1% MeOH/CH_2Cl_2), yield = 61%. Mp = 141 – 144 °C, R_f = 0.45 (5% MeOH/CH_2Cl_2). ^{1}H NMR (400 MHz, $CDCl_3$, 25 °C), δ (ppm) = 7.58 (d, 1H, J = 15.7 Hz, =CHC_{ar}), 7.38 (dd, 1H, J = 8.4 Hz, 1.7 Hz, H_{ar}), 7.33 (d, 1H, J = 1.6 Hz, H_{ar}), 7.28 (s, 1H, =CHN), 7.21 (d, 1H, J = 8.4 Hz, H_{ar}), 6.63 (br s, 1H, NH),

6.37 (d, 1H, J = 15.7 Hz, =CHCO), 4.52 (t, 2H, J = 5.9 Hz, CH_2-triazole), 3.91 (q, 2H, J = 5.9 Hz, NHC**H**$_2$), 2.74 (m, 1H, CH-triazole), 2.32 (s, 3H, CH_3COO), 2.31 (s, 3H, CH_3COO), 2.05 (m, 2H, cyclohexyl), 1.82-1.72 (m, 2H, cyclohexyl), 1.45-1.24 (m, 6H, cyclohexyl). ^{13}C NMR (101 MHz, $CDCl_3$, 25 °C), δ (ppm) = 168.13, 168.09, 165.91, 153.78, 143.10, 142.37, 139.70, 133.62, 126.24, 123.86, 122.46, 121.33, 120.54, 49.33, 39.47, 35.21, 32.98, 26.09, 25.98, 20.67, 20.62. HRMS m/z calc. for $C_{23}H_{28}N_4O_5$ + (H^+) : 441.2132; detected : 441.2126.

*(E)-4-(3-((2-(4-(Cyclohex-1-en-1-yl)-1H-1,2,3-triazol-1-yl)ethyl)amino)-3-oxoprop-1-en-1-yl)-1,2-phenylene diacetate (**14d**)*

Following general procedure IIB with azide **12** and 1-ethynylcyclohex-1-ène**,** compound **14d** was obtained as a white solid after silica gel circular chromatography (0 – 1% MeOH/CH_2Cl_2), yield = 70%. Mp = 160 – 162 °C, R_f = 0.28 (5% MeOH/CH_2Cl_2). ^{1}H NMR (400 MHz, $CDCl_3$, 25 °C), δ (ppm) = 7.57 (d, 1H, J = 15.7 Hz, =CHC_{ar}), 7.42 (s, 1H, =CHN), 7.37 (d, 1H, J = 8.4 Hz, H_{ar}), 7.33 (s, 1H, H_{ar}), 7.20 (d, 1H, J = 8.4 Hz, H_{ar}), 6.69 (br s, 1H, NH), 6.48 (m, 1H, CH=C-triazole), 6.37 (d, 1H, J = 15.7 Hz, =CHCO), 4.53 (t, 2H, J = 5.4 Hz, CH_2-triazole), 3.92 (q, 2H, J = 5.4 Hz, NHC**H**$_2$), 2.36 (m, 2H, cyclohexenyl), 2.32 (s, 3H, CH_3COO), 2.31 (s, 3H, CH_3COO), 2.19 (m, 2H, cyclohexenyl), 1.78-1.66 (m, 4H, cyclohexenyl). ^{13}C NMR (101 MHz, $CDCl_3$, 25 °C), δ (ppm) = 168.12, 168.09, 165.97, 149.48, 143.10, 142.36, 139.68, 133.62, 127.05, 126.24, 125.35, 123.85, 122.47, 121.34, 119.49, 49.45, 39.59, 26.34, 25.26, 22.40, 22.14, 20.67, 20.62. HRMS m/z calc. for $C_{23}H_{26}N_4O_5$ + (H^+) : 439.1976; detected : 439.1976

*(E)-4-(3-oxo-3-((2-(4-Phenyl-1H-1,2,3-triazol-1-yl)ethyl)amino)prop-1-en-1-yl)-1,2-phenylene diacetate (**14e**)*

Following general procedure IIB with azide **12** and ethynylbenzene**,** compound **14e** was obtained as a white solid after silica gel circular chromatography (0 – 1% MeOH/CH_2Cl_2), yield = 28%. Mp = 142 – 148 °C, R_f = 0.38 (5% MeOH/CH_2Cl_2). ^{1}H NMR (400 MHz, $CDCl_3$, 25 °C), δ (ppm) = 7.77 – 7.74 (m, 3H, H_{ar} + =CHN), 7.57 (d, 1H, J = 15.7 Hz, =CHC_{ar}), 7.41 – 7.30 (m, 5H, H_{ar}), 7.17 (d, 1H, J = 8.4 Hz, H_{ar}), 6.86 (br s, 1H, NH), 6.39 (d, 1H, J = 15.7 Hz, =CHCO), 4.60 (m,

2H, CH_2-triazole), 3.94 (m, 2H, C**H₂**NH), 2.30 (s, 3H, CH_3CO), 2.29 (s, 3H, CH_3CO). ^{13}C NMR (101 MHz, $CDCl_3$, 25 °C), δ (ppm) = 168.13, 168.10, 166.11, 147.73, 143.09, 142.33, 139.76, 133.57, 130.16, 128.91, 128.34, 126.28, 125.63, 123.83, 122.47, 121.30, 120.81, 49.67, 39.66, 20.66, 20.60. HRMS m/z calc. For. $C_{23}H_{22}N_4O_5$ + (H^+) : 435.1663; detected : 435.1660.

*(E)-4-(3-oxo-3-((2-(4-(p-Tolyl)-1H-1,2,3-triazol-1-yl)ethyl)amino)prop-1-en-1-yl)-1,2-phenylene diacetate (**14f**)*

Following general procedure IIB with azide **12** and 1-ethynyl-4-methylbenzene**,** compound **14f** was obtained as a white solid after silica gel circular chromatography (0 – 1.4% $MeOH/CH_2Cl_2$), yield = 61%. Mp = 177 – 178 °C, R_f = 0.48 (AcOEt). ^1H NMR (400 MHz, $CDCl_3$, 25 °C), δ (ppm) = 7.71 (s, 1H, =CHN), 7.62 (d, 2H, J = 8.0 Hz, H_{ar}), 7.57 (d, 1H, J = 15.7 Hz, =CHC), 7.34 - 7.29 (m, 2H, H_{ar}), 7.20 - 7.15 (m, 3H, H_{ar}), 6.95 (br s, 1H, NH), 6.40 (d, 1H, J = 15.7 Hz, =CHCO), 4.58 (t, 2H, J = 5.4 Hz, CH_2-triazole), 3.92 (q, 2H, J = 5.4 Hz, NHC**H₂**), 2.37 (s, 3H, CH_3Ph), 2.30 (s, 3H, CH_3COO), 2.29 (s, 3H, CH_3COO). ^{13}C NMR (101 MHz, $CDCl_3$, 25 °C), δ (ppm) = 168.13, 168.10, 166.11, 147.79, 143.06, 142.32, 139.65, 138.21, 133.62, 129.56, 127.33, 126.26, 125.52, 123.81, 122.48, 121.39, 120.48, 49.62, 39.66, 21.27, 20.66, 20.59. HRMS m/z calc. for $C_{24}H_{24}N_4O_5$ + (H^+): 449.1819; detected: 449.1820.

*(E)-4-(3-((2-(4-(4-Fluorophenyl)-1H-1,2,3-triazol-1-yl)ethyl)amino)-3-oxoprop-1-en-1-yl)-1,2-phenylene diacetate (**14g**)*

Following general procedure IIB with azide **12** and 1-ethynyl-4-methylbenzene**,** compound **14g** was obtained as a white solid after silica gel circular chromatography (0 – 1.3% $MeOH/CH_2Cl_2$), yield = 58%. Mp = 165 °C, R_f = 0.50 (5% $MeOH/CH_2Cl_2$). ^1H NMR (400 MHz, $CDCl_3$, 25 °C), δ (ppm) = 7.79-7.75 (m, 3H, =CHN + H_{ar}), 7.59 (d, 1H, J = 15.7 Hz, =CHC_{ar}), 7.36 (dd, 1H, J = 8.4 Hz, 2.0 Hz, H_{ar}), 7.32 (d, 1H, J = 2.0 Hz, H_{ar}), 7.20 (d, 1H, J = 8.4 Hz, H_{ar}), 7.15 - 7.10 (m, 2H, H_{ar}), 6.39 (t, 1H, J = 5.7 Hz, NH), 6.34 (d, 1H, J = 15.7 Hz, =CHCO), 4.62 (t, 2H, J = 5.7 Hz, CH_2-triazole), 3.98 (q, 2H, J = 5.7 Hz, NHC**H₂**), 2.32 (s, 3H, CH_3COO), 2.31 (s, 3H, CH-$_3$COO). ^{13}C NMR (101 MHz, $CDCl_3$, 25 °C), δ (ppm) = 168.11, 168.05, 165.92, 163.99, 161.52,

147.03, 143.20, 142.39, 140.07, 133.44, 127.47, 127.39, 126.47, 126.44, 126.28, 123.89, 122.46, 120.99, 120.47, 116.03, 115.82, 49.64, 39.58, 20.65, 20.61. HRMS m/z calc. for $C_{23}H_{21}FN_4O_5$ + (H^+): 453.1569; detected: 453.1567.

*(E)-4-(3-((2-(4-(4-Formylphenyl)-1H-1,2,3-triazol-1-yl)ethyl)amino)-3-oxoprop-1-en-1-yl)-1,2-phenylene diacetate (**14h**)*

Following general procedure IIB with azide **12** and 1-ethynyl-4-methylbenzene**,** compound **14h** was obtained as a white solid after silica gel circular chromatography (0 – 1.3% MeOH/CH_2Cl_2), yield = 51%. Mp = 181 – 182 °C, R_f = 0.33 (5% MeOH/CH_2Cl_2). ^{1}H NMR (400 MHz, DMSO-d_6, 25 °C), δ (ppm) = 10.02 (s, 1H, CHO), 8.81 (s, 1H, =CHN), 8.35 (t, 1H, J = 5.7 Hz, NH), 8.10 (d, 2H, J = 8.2 Hz, H_{ar}), 7.99 (d, 2H, J = 8.2 Hz, H_{ar}), 7.51-7.48 (m, 2H, H_{ar}), 7.43 (d, 1H, J = 15.7 Hz, =CHC_{ar}), 7.31 (d, 1H, J = 8.1 Hz, H_{ar}), 6.58 (d, 1H, J = 15.7 Hz, =CHCO), 4.58 (t, 2H, J = 5.7 Hz, CH_2-triazole), 3.71 (q, 2H, J = 5.7 Hz, NHC**H_2**), 2.30 (s, 3H, CH_3COO), 2.29 (s, 3H, CH_3COO). ^{13}C NMR (101 MHz, DMSO-d_6, 25 °C), δ (ppm) = 192.99, 168.66, 168.62, 165.63, 145.67, 143.21, 142.75, 137.93, 136.93, 135.78, 134.09, 130.80, 126.43, 125.96, 124.61, 123.82, 123.23, 122.82, 49.78, 39.29, 20.82, 20.81. HRMS m/z calc. for $C_{24}H_{22}N_4O_6$ + (H^+): 463.1612; detected: 463.1619.

*(E)-2-(4-Propyl-1H-1,2,3-triazol-1-yl)ethyl 3-(3,4-dihydroxyphenyl)acrylate (**15b**)*

Following general procedure III with acetylated caffeoyl derivative **13b,** compound **15b** was obtained as a white powder, yield = 43%. Mp = 180 – 181 °C (dec.), R_f = 0.41 (5% MeOH/CH_2Cl_2). ^{1}H NMR (400 MHz, DMSO-d_6, 25 °C), δ (ppm) = 9.66 (br s, 1H, OH), 9.24 (br s, 1H, OH), 7.92 (s, 1H =CHN), 7.44 (d, 1H, J = 15.9 Hz, =CHC_{ar}), 7.03 (s, 1H, H_{ar}), 6.99 (d, 1H, J = 8.3 Hz, H_{ar}), 6.76 (d, 1H, J = 8.3 Hz, H_{ar}), 6.22 (d, 1H, J = 15.9 Hz, =CHCO), 4.64 (t, 2H, J = 5.0 Hz, OCH_2), 4.49 (t, 2H, J = 5.0 Hz, CH_2N), 2.59 (t, 2H, J = 7.5 Hz, **CH_2**CH_2CH_3), 1.60 (sext., 2H, J = 7.4 Hz, CH_2**CH_2**CH_3), 0.88 (t, 3H, J = 7.4 Hz, CH_3). ^{13}C NMR (101 MHz, DMSO-d_6, 25 °C), δ (ppm) = 166.57, 149.03, 147.24, 146.20, 146.04, 125.81, 122.78, 121.95,

116.18, 115.36, 113.73, 62.75, 48.90, 27.48, 22.72, 14.01. HRMS m/z calc. for $C_{16}H_{19}N_3O_4$ + (H^+) : 318.1448; detected : 318.1455.

*(E)-2-(4-Cyclohexyl-1H-1,2,3-triazol-1-yl)ethyl 3-(3,4-dihydroxyphenyl)acrylate (**15c**)*

Following general procedure III with acetylated caffeoyl derivative **13c,** compound **15c** was obtained as a white powder, yield = 80%. Mp = 191 °C (dec.), R_f = 0.33 (5% MeOH/CH_2Cl_2). ^{1}H NMR (400 MHz, DMSO-d_6, 25 °C), δ (ppm) = 9.65 (br s, 1H, OH), 9.15 (br s, 1H, OH), 7.90 (s, 1H, =CHN), 7.44 (d, 1H, J = 15.9 Hz, =CHC_{ar}), 7.04 (s, 1H, H_{ar}), 6.99 (d, 1H, J = 8.2 Hz, H_{ar}), 6.76 (d, 1H, J = 8.2 Hz, H_{ar}), 6.23 (d, 1H, J = 15.9 Hz, =CHCO), 4.63 (t, 2H, J = 4.8 Hz, OCH_2), 4.48 (t, 2H, J = 4.8 Hz, CH_2N), 2.70 – 2.61 (m, 1H, CH-triazole), 2.33 (s, 3H, CH_3COO), 2.32 (s, 3H, CH_3COO), 1.98 – 1.88 (m, 2H, cyclohexyl), 1.76 – 1.62 (m, 4H, cyclohexyl), 1.41 – 1.30 (m, 4H, cyclohexyl); ^{13}C NMR (101 MHz, DMSO-d_6, 25 °C), δ (ppm) = 166.56, 152.64, 149.03, 146.21, 146.04, 125.82, 121.95, 121.54, 116.18, 115.37, 113.75, 62.72, 48.90, 35.01, 33.02, 26.08, 25.99. HRMS m/z calc. for $C_{19}H_{23}N_3O_4$ + (H^+) : 358.1761; detected : 358.1760.

*(E)-2-(4-(Cyclohex-1-en-1-yl)-1H-1,2,3-triazol-1-yl)ethyl 3-(3,4-dihydroxyphenyl)acrylate (**15d)***

Following general procedure III with acetylated caffeoyl derivative **13d,** compound **15d** was obtained as a white powder, yield = 75%. Mp = 219 - 220 °C (dec.), R_f = 0.35 (5% MeOH/CH_2Cl_2). ^{1}H NMR (400 MHz, DMSO-d_6, 25 °C), δ (ppm) = 9.65 (br s, 1H, OH), 9.16 (br s, 1H, OH), 8.15 (s, 1H, =CHN), 7.45 (d, 1H, J = 15.9 Hz, =CHC_{ar}), 7.04 (d, 1H, J = 1.8 Hz, H_{ar}), 7.00 (dd, 1H, J = 8.2 Hz, 1.8 Hz, H_{ar}), 6.76 (d, 1H, J = 8.2 Hz, H_{ar}), 6.40 - 6.38 (m, 1H, CH=C-triazole), 6.23 (d, 1H, J = 15.9 Hz, =CHCO), 2.32 – 2.38 (m, 2H, cyclohexenyl), 2.15 – 2.13 (m, 2H, cyclohexenyl), 1.72 - 1.66 (m, 2H, cyclohexenyl), 1.63 - 1.59 (m, 2H, cyclohexenyl), ^{13}C NMR (101 MHz, DMSO-d_6, 25 °C), δ (ppm) = 166.60, 149.03, 148.57, 146.25, 146.04, 127.91, 125.81, 123.87, 121.97, 120.78, 116.18, 115.38, 113.72, 62.70, 49.06, 26.26, 25.11, 22.48, 22.33. HRMS m/z calc. for $C_{19}H_{21}N_3O_4$ + (H^+) : 356.1605; detected : 356.1611.

*(E)-2-(4-Phenyl-1H-1,2,3-triazol-1-yl)ethyl 3-(3,4-dihydroxyphenyl)acrylate (**15e**)*

Following general procedure III with acetylated caffeoyl derivative **13e,** compound **15e** was obtained as a light brown powder, yield = 51%. Mp = 193 - 195 °C (dec.), R_f = 0.32 (5% MeOH/CH_2Cl_2). ^{1}H NMR (400 MHz, DMSO-d_6, 25 °C), δ (ppm) = 9.65 (br s, 1H, OH), 9.17 (br s, 1H, OH), 8.68 (s, 1H, =CHN), 7.67 (d, 2H, J = 7.4 Hz, H_{ar}), 7.49-7.43 (m, 3H, H_{ar} + =CHC_{ar}), 7.34 (m, 1H, H_{ar}), 7.05 (s, 1H, H_{ar}), 6.99 (d, 1H, J = 8.2 Hz, H_{ar}), 6.76 (d, 1H, J = 8.2 Hz, H_{ar}), 6.25 (d, 1H, J = 15.9 Hz, =CHCO), 4.62 (t, 2H, J = 4.7 Hz, OCH_2), 4.57 (t, 2H, J = 4.7 Hz, CH_2N). ^{13}C NMR (101 MHz, DMSO-d_6, 25 °C), δ (ppm) = 166.64, 149.04, 146.87, 146.30, 146.04, 131.19, 129.38, 128.34, 125.81, 125.61, 122.34, 121.99, 116.19, 115.40, 113.70, 62.67, 49.36. HRMS m/z calc. for $C_{19}H_{17}N_3O_4$ + (H^+) : 352.1292; detected : 352.1302.

*(E)-2-(4-(p-Tolyl)-1H-1,2,3-triazol-1-yl)ethyl 3-(3,4-dihydroxyphenyl)acrylate (**15f**)*

Following general procedure III with acetylated caffeoyl derivative **13f,** compound **15f** was obtained as a white powder, yield = 79%. Mp = 181 - 182 °C (dec.), R_f = 0.26 (5% MeOH/CH_2Cl_2). ^{1}H NMR (400 MHz, DMSO-d_6, 25 °C), δ (ppm) = 9.64 (br s, 1H, OH), 9.156 (br s, 1H, OH), 8.60 (s, 1H, =CHN), 7.74 (d, 2H, J = 8.0 Hz , H_{ar}), 7.47 (d, 1H, J = 15.9 Hz, =CHC_{ar}), 7.26 (d, 2H, J = 8.0 Hz, H_{ar}), 7.04 (s, 1H, H_{ar}), 6.99 (d, 1H, J = 8.2 Hz, H_{ar}), 6.75 (d, 1H, J = 8.2 Hz, H_{ar}), 6.25 (d, 1H, J =15.9 Hz, =CHCO), 4.74 (t, 2H, J = 4.7 Hz, OCH_2), 4.56 (t, 2H, J = 4.7 Hz, CH_2N), 2.33 (s, 3H, C_{ar}-CH_3). ^{13}C NMR (101 MHz, DMSO-d_6, 25 °C), δ (ppm) = 166.64, 149.03, 146.94, 146.29, 146.03, 137.63, 129.92, 128.42, 125.82, 125.55, 122.00, 121.89, 116.17, 115.38, 113.72, 62.67, 49.31, 21.30. HRMS m/z calc. for $C_{20}H_{19}N_3O_4$ + (H^+) : 366.1448; detected : 366.1454.

*(E)-2-(4-(4-Fluorophenyl)-1H-1,2,3-triazol-1-yl)ethyl 3-(3,4-dihydroxyphenyl)acrylate (**15g**)*

Following general procedure III with acetylated caffeoyl derivative **13g** compound **15g** was obtained as a white powder, yield = 63%. Mp = 238 - 240 °C (dec.), R_f = 0.28 (5% MeOH/CH_2Cl_2). ^{1}H NMR (400 MHz, DMSO-d_6, 25 °C), δ (ppm) = 9.65 (br s, 1H, OH), 9.15 (br s, 1H, OH), 8.66 (s, 1H, =CHN), 7.90 (t, 2H, J = 8.6 Hz, H_{ar}), 7.47 (d, 1H, J = 15.9 Hz, =CHC_{ar}),

7.29 (d, 2H, J = 8.6 Hz, H_{ar}), 7.04 (s, 1H, H_{ar}), 6.99 (d, 1H, J = 8.2 Hz, H_{ar}), 6.75 (d, 1H, J = 8.2 Hz, H_{ar}), 6.25 (d, 1H, J = 16.0 Hz, =CHCO), 4.75 (t, 2H, J = 4.6 Hz, OCH_2), 4.56 (t, 2H, J = 4.6 Hz, CH_2N), ^{13}C NMR (101 MHz, DMSO-d_6, 25 °C), δ (ppm) = 166.64, 163.44, 161.01, 149.04, 146.29, 146.03, 127.75, 127.67, 127.58, 125.82, 122.23, 122.00, 116.42, 116.20, 116.17, 115.39, 113.71, 62.65, 49.39. HRMS m/z calc. for $C_{19}H_{16}FN_3O_4$ + (H^+) : 370.1198; detected : 370.1201.

*(E)-2-(4-(4-Formylphenyl)-1H-1,2,3-triazol-1-yl)ethyl 3-(3,4-dihydroxyphenyl)acrylate (**15h**)*

Following general procedure III with acetylated caffeoyl derivative **13h,** compound **15h** was obtained as a beige powder, yield = 61%. Mp = 220 - 230 °C (dec.), R_f = 0.32 (5% MeOH/CH_2Cl_2). ^1H NMR (400 MHz, DMSO-d_6, 25 °C), δ (ppm) = 10.019 (s, 1H, CHO), 9.65 (br s, 1H, OH), 9.15 (br s, 1H, OH), 8.88 (s, 1H, =CHN), 8.10 (d, 2H, J = 8.2 Hz, H_{ar}), 8.00 (d, 2H, J = 8.2 Hz, H_{ar}), 7.47 (d, 1H, J = 15.8 Hz, =CHC_{ar}), 7.04 (s, 1H, H_{ar}), 6.99 (d, 1H, J = 8.2 Hz, H_{ar}), 6.75 (d, 1H, J = 8.2 Hz, H_{ar}), 6.23 (d, 1H, J = 15.9 Hz, =CHCO), 4.79 (t, 2H, J = 4.7 Hz, OCH_2), 4.58 (t, 2H, J = 4.7 Hz, CH_2N), ^{13}C NMR (101 MHz, DMSO-d_6, 25 °C), δ (ppm) = 192.99, 166.63, 149.04, 146.32, 146.03, 145.79, 136.79, 135.84, 130.80, 126.01, 125.81, 123.94, 122.00, 116.17, 115.39, 113.69, 62.60, 49.54. HRMS m/z calc. for $C_{20}H_{17}N_3O_5$ + (H^+) : 380.1241; detected : 380.1250.

*(E)-N-(2-(1H-1,2,3-Triazol-1-yl)ethyl)-3-(3,4-dihydroxyphenyl)acrylamide (**16a**)*

Following general procedure III with acetylated caffeoyl derivative **14a,** compound **16a** was obtained as a white powder, yield = 54%. Mp = 163 - 165 °C, R_f = 0.15 (7.5% MeOH/CH_2Cl_2). ^1H NMR (400 MHz, DMSO-d_6, 25 °C), δ (ppm) = 9.41 (br s, 1H, OH), 9.16 (br s, 1H, OH), 8.19 (br s, 1H, NH), 8.12 (s, 1H, =CHN), 7.72 (s, 1H, =CHN), 7.24 (d, 1H, J = 15.7 Hz, =CHC_{ar}), 6.94 (s, 1H, H_{ar}), 6.83 (d, 1H, J = 8.1 Hz, H_{ar}), 6.75 (d, 1H, J = 8.1 Hz, H_{ar}), 6.28 (d, 1H, J = 15.7 Hz, =CHCO), 4.50 (t, 2H, J = 5.9 Hz, CH-triazole), 3.61 (q, 2H, J = 5.9 Hz, NHC**H**$_2$). ^{13}C NMR (101 MHz, DMSO-d_6, 25 °C), δ (ppm) = 166.27, 147.88, 146.00, 140.02, 133.67, 126.65, 125.51, 120.95, 118.37, 116.22, 114.32, 49.17, 39.36. HRMS m/z calc. for $C_{13}H_{14}N_4O_3$ + (H^+): 275.1139; detected : 274.1146

*(E)-3-(3,4-Dihydroxyphenyl)-N-(2-(4-propyl-1H-1,2,3-triazol-1-yl)ethyl)acrylamide (**16b**)*

Following general procedure III with acetylated caffeoyl derivative **14b,** compound **16b** was obtained as a grey solide, yield = 55%. Mp = 163 – 165 °C, R_f = 0.33% (7.5% MeOH/CH_2Cl_2). ^{1}H NMR (400 MHz, MeOD, 25 °C), δ (ppm) = 7.73 (s, 1H, =CHN), 7.39 (d, 1H, J = 15.7 Hz, =CHC_{ar}), 7.00 (d, 1H, J = 1.7 Hz, H_{ar}), 6.90 (dd, 1H, J = 8.4 Hz, 1.8 Hz, H_{ar}), 6.77 (d, 1H, J = 8.4 Hz, H_{ar}), 6.31 (d, 1H, J = 15.7 Hz, CHCO), 4.56 (t, 2H, J = 5.9 Hz, CH_2-triazole), 3.77 (t, 2H, J = 5.9 Hz, C**H**$_2$NH), 2.68 (t, 2H, J = 7.6 Hz, C**H**$_2$$CH_2CH_3$), 1.69 (m, 2H, CH_2C**H**$_2$$CH_3$), 0.97 (t, 3H, J = 7.4 Hz, CH_3). ^{13}C NMR (101 MHz, MeOD, 25 °C), δ (ppm) =168.19, 149.67, 147.52, 145.34, 141.36, 126.70, 122.23, 120.79, 116.37, 115.03, 113.62, 49.02, 39.25, 26.87, 22.35, 12.55. HRMS m/z calc. for $C_{16}H_{20}N_4O_3$ + (H^+) : 317.1608; detected : 317.1612.

*(E)-N-(2-(4-Cyclohexyl-1H-1,2,3-triazol-1-yl)ethyl)-3-(3,4-dihydroxyphenyl)acrylamide (**16c**)*

Following general procedure III with acetylated caffeoyl derivative **14c,** compound **16c** was obtained as a beige solide, yield = 81%. Mp = 183 - 184, R_f = 0.42% (7.5% MeOH/CH_2Cl_2). ^{1}H NMR (400 MHz, DMSO-d$_6$, 25 °C), δ (ppm) = 9.39 (br s, 1H, OH), 9.14 (br s, 1H, OH), 8.16 (m, 1H, NH), 7.81 (s, 1H, =CHN), 7.24 (d, 1H, J = 15.7 Hz, =CHC_{ar}), 6.93 (d, 1H, J = 1.9 Hz, H_{ar}), 6.83 (dd, 1H, J = 8.2 Hz, 1.9 Hz, H_{ar}), 6.74 (d, 1H, J = 8.2 Hz, H_{ar}), 6.29 (d, 1H, J = 15.7 Hz, =CHCO), 4.41 (t, 2H, J = 6.0 Hz, CH_2-triazole), 3.59 (q, 2H, J = 6.0 Hz, NHC**H**$_2$), 2.63 (m, 1H, CH-triazole), 1.98 - 1.91 (m, 2H, cyclohexyl), 1.73 - 1.67 (m, 2H, cyclohexyl), 1.41 - 1.19 (m, 6H, cyclohexyl). ^{13}C NMR (101 MHz, DMSO-d$_6$, 25 °C), δ (ppm) = 166.28, 152.45, 147.86, 145.99, 140.00, 126.68, 121.31, 120.96, 118.41, 116.19, 114.28, 49.15, 39.47, 35.05, 33.00, 26.11, 26.01. HRMS m/z calc. for $C_{19}H_{24}N_4O_3$ + (H^+): 357.1912; detected : 357.1930.

*(E)-N-(2-(4-(Cyclohex-1-en-1-yl)-1H-1,2,3-triazol-1-yl)ethyl)-3-(3,4-dihydroxyphenyl)acrylamide (**16d**)*

Following general procedure III with acetylated caffeoyl derivative **14d,** compound **16d** was obtained as a beige solide, yield = 80%. Mp = 168 – 169 °C, R_f = 0.40% (7.5% MeOH/CH_2Cl_2). ^{1}H NMR (400 MHz, MeOD, 25 °C), δ (ppm) = 7.90 (s, 1H, =CHN), 7.39 (d, 1H, J = 15.7 Hz,

$=CHC_{ar}$), 7.00 (s, 1H, H_{ar}), 6.91 (d, 1H, J = 8.0 Hz, H_{ar}), 6.77 (d, 1H, J = 8.0 Hz, H_{ar}), 6.44 (br s, 1H, CH=C-triazole), 6.31 (d, 1H, J = 15.7 Hz, =CHCO), 4.56 (t, 2H, J = 5.5 Hz, CH_2-triazole), 3.77 (t, 2H, J = 5.5 Hz, NHC**H**$_2$), 2.38 (m, 2H, cyclohexenyl), 2.31 (m, 2H, cyclohexenyl), 1.78 (m, 2H, cyclohexenyl), 1.69 (m, 2H, cyclohexenyl). ^{13}C NMR (101 MHz, MeOD, 25 °C), δ (ppm) = 168.24, 149.10, 147.52, 145.33, 141.38, 127.01, 126.71, 124.63, 120.82, 119.92, 116.38, 115.03, 113.63, 49.14, 39.26, 25.91, 24.82, 22.16, 21.87. HRMS m/z calc. for $C_{19}H_{22}N_4O_3$ + (H^+) : 355.1765; detected : 355.1772

*(E)-3-(3,4-Dihydroxyphenyl)-N-(2-(4-phenyl-1H-1,2,3-triazol-1-yl)ethyl)acrylamide (**16e**)*

Following general procedure III with acetylated caffeoyl derivative **14e,** compound **16e** was obtained as a beige solide, yield = 73%. Mp = 208 °C (dec.), R_f = 0.40% (10% MeOH/CH_2Cl_2). ^1H NMR (400 MHz, DMSO-d_6, 25 °C), δ (ppm) = 9.39 (br s, 1H, OH), 9.14 (br s, 1H, OH), 8.59 (s, 1H, =CHN), 8.21 (br s, 1H, NH), 7.84 (m, 2H, H_{ar}), 7.43 (m, 2H, H_{ar}), 7.31 (m, 1H, H_{ar}), 7.25 (d, 1H, J = 15.7 Hz, $=CHC_{ar}$), 6.93 (s, 1H, H_{ar}), 6.83 (d, 1H, J = 7.3 Hz, H_{ar}), 6.73 (d, 1H, J = 7.3 Hz, H_{ar}), 6.29 (d, 1H, J = 15.7 Hz, =CHCO), 4.52 (m, 2H, CH_2-triazole), 3.68 (m, 2H, NHC**H**$_2$). ^{13}C NMR (101 MHz, DMSO-d_6, 25 °C), δ (ppm) = 166.34, 147.88, 146.73, 145.99, 140.07, 131.31, 129.36, 128.26, 126.65, 125.57, 122.18, 120.97, 118.37, 116.19, 114.31, 49.69, 39.29. HRMS m/z calc. for $C_{19}H_{18}N_4O_3$ + (H^+) : 351.1452; detected : 351.1447.

*(E)-3-(3,4-Dihydroxyphenyl)-N-(2-(4-(p-tolyl)-1H-1,2,3-triazol-1-yl)ethyl)acrylamide (**16f**)*

Following general procedure III with acetylated caffeoyl derivative **14f,** compound **16f** was obtained as a beige solide, yield = 66%. Mp = 220 °C (dec.), R_f = 0.34% (7.5% MeOH/CH_2Cl_2). ^1H NMR (400 MHz, DMSO-d_6, 25 °C), δ (ppm) = 9.39 (s, 1H, OH), 9.13 (s, 1H, OH), 8.52 (s, 1H, =CHN), 8.21 (t, 1H, J = 5.7 Hz, NH), 7.73 (d, 2H, J = 8.0 Hz, H_{ar}), 7.27-7.23 (m, 3H, H_{ar} + $=CHC_{ar}$), 6.93 (d, 1H, J = 1.6 Hz, H_{ar}), 6.83 (dd, 1H, J = 8.2 Hz, 1.6 Hz, H_{ar}), 6.73 (d, 1H, J = 8.2 Hz, H_{ar}), 6.29 (d, 1H, J = 15.7 Hz, =CHCO), 4.51 (t, 2H, J = 5.7 Hz, CH_2-triazole), 3.66 (q, 2H, J = 5.7 Hz, NHC**H**$_2$), 2.33 (s, 3H, CH_3). ^{13}C NMR (101 MHz, DMSO-d_6, 25 °C), δ (ppm) = 166.33, 147.87, 146.79, 145.98, 140.06, 137.52, 129.90, 128.55, 126.66, 125.52, 121.74, 120.96,

118.39, 116.20, 114.32, 49.64, 39.32, 21.30. HRMS m/z calc. for $C_{20}H_{20}N_4O_3$ + (H^+) : 365.1608; detected : 365.1614.

*(E)-3-(3,4-Dihydroxyphenyl)-N-(2-(4-(4-fluorophenyl)-1H-1,2,3-triazol-1-yl)ethyl)acrylamide **(16g)***

Following general procedure III with acetylated caffeoyl derivative **14g,** compound **16g** was obtained as a light yellow solid, yield = 68%. Mp = 226 °C (dec.), R_f = 0.36% (7.5% MeOH/CH_2Cl_2). ^{1}H NMR (400 MHz, DMSO-d_6, 25 °C), δ (ppm) = 9.39 (s, 1H, OH), 9.13 (s, 1H, OH), 8.58 (s, 1H, =CHN), 8.20 (m, 1H, NH), 7.88 (m, 2H, H_{ar}), 7.31-7.23 (m, 3H, H_{ar} + =CHC_{ar}), 6.93 (d, 1H, J = 1.8 Hz, H_{ar}), 6.83 (dd, 1H, J = 8.2 Hz, 1.8 Hz, H_{ar}), 6.74 (d, 1H, J = 8.2 Hz, H_{ar}), 6.28 (d, 1H, J = 15.7 Hz, =CHCO), 4.52 (t, 2H, J = 5.7 Hz, CH_2-triazole), 3.66 (q, 2H, J = 5.7 Hz, NHC**H_2**). ^{13}C NMR (101 MHz, DMSO-d_6, 25 °C), δ (ppm) = 166.34, 163.39, 160.96, 147.88, 145.99, 145.88, 140.07, 127.90, 127.61, 127.53, 126.65, 122.10, 120.98, 118.36, 116.41, 116.19, 114.30, 49.72, 39.36. HRMS m/z calc. for $C_{19}H_{17}FN_4O_3$ + (H^+) : 369.1357; detected : 369.1362.

*(E)-3-(3,4-Dihydroxyphenyl)-N-(2-(4-(4-formylphenyl)-1H-1,2,3-triazol-1-yl)ethyl)acrylamide **(16h)***

Following general procedure III with acetylated caffeoyl derivative **14h,** compound **16h** was obtained as a white solid, yield = 58%. Mp = 228 – 230 °C (dec.), R_f = 0.20% (5% MeOH/CH_2Cl_2). ^{1}H NMR (400 MHz, DMSO-d_6, 25 °C), δ (ppm) = 10.02 (s, 1H, CHO), 9.40 (br s, 1H, OH), 9.14 (br s, 1H, OH), 8.80 (s, 1H, =CHN), 8.21 (t, 1H, J = 5.7 Hz, NH), 8.09 (d, 2H, J = 8.2 Hz, H_{ar}), 7.99 (d, 2H, J = 8.2 Hz, H_{ar}), 7.25 (d, 1H, J = 15.7 Hz, =CHC_{ar}), 6.93 (s, 1H, H_{ar}), 6.83 (d, 1H, J = 8.1 Hz, H_{ar}), 6.73 (d, 1H, J = 8.1 Hz, H_{ar}), 6.28 (d, 1H, J = 15.7 Hz, =CHCO), 4.55 (t, 2H, J = 5.7 Hz, CH_2-triazole), 3.69 (q, 2H, J = 5.7 Hz, NHC**H_2**). ^{13}C NMR (101 MHz, DMSO-d_6, 25 °C), δ (ppm) = 193.00, 163.35, 147.89, 145.99, 145.65, 140.09, 136.94, 135.78, 130.80, 126.64, 125.96, 123.82, 120.98, 118.35, 116.20, 114.31, 49.89, 39.47. HRMS m/z calc. for $C_{20}H_{18}N_4O_4$ + (H^+) : 379.1401; detected : 379.1405.
